# Tracing genomic instability in induced mesenchymal stromal cell manufacture: an integration-free transfection approach

**DOI:** 10.1038/s12276-025-01439-8

**Published:** 2025-04-14

**Authors:** Jong-Mi Lee, Chae Yeon Lee, Binna Seol, Chan Kwon Jung, Yonggoo Kim, Dain Kang, Haein Yu, Yuna Hong, Cho Lok Song, Yee Sook Cho, Myungshin Kim

**Affiliations:** 1 Catholic Genetic Laboratory Center, Seoul St. Mary’s Hospital, College of Medicine, The Catholic University of Korea, Seoul, Republic of Korea; 2 Department of Laboratory Medicine, College of Medicine, The Catholic University of Korea, Seoul, Republic of Korea; 3 Department of Medical Sciences, Graduate School of The Catholic University of Korea, Seoul, Republic of Korea; 4 Stem Cell Research Laboratory, Immunotherapy Research Center, Korea Research Institute of Bioscience and Biotechnology (KRIBB), Daejeon, Republic of Korea; 5 Department of Hospital Pathology, College of Medicine, The Catholic University of Korea, Seoul, Republic of Korea; 6 Department of Bioscience, KRIBB School, University of Science and Technology, Daejeon, Republic of Korea

**Keywords:** Genomic instability, Reprogramming

## Abstract

Here we systematically investigated genomic alterations from the initiation of induced pluripotent stem (iPS) cell generation to induced mesenchymal stromal/stem cell differentiation. We observed a total of ten copy number alterations (CNAs) and five single-nucleotide variations (SNVs) during the phases of reprogramming, differentiation and passaging. We identified a higher frequency of CNAs and SNVs in iPS cells generated using the Sendai virus (SV) method compared with those generated with episomal vectors (Epi). Specifically, all SV-iPS cell lines exhibited CNAs during the reprogramming phase, while only 40% of Epi-iPS cells showed such alterations. Additionally, SNVs were observed exclusively in SV-derived cells during passaging and differentiation, with no SNVs detected in Epi-derived lines. Gene expression analysis revealed upregulation of chromosomal instability-related genes in late-passage SV-iPSCs, further indicating increased genomic instability. Notably, *TP53* mutations were identified, underscoring the vulnerability of the gene and the critical need for careful genomic scrutiny when preparing iPS cells and derived cell lines.

## Genomic instability in induced pluripotent stem cells revealed

This study explores the potential of using induced pluripotent stem (iPS) cells to create mesenchymal stem (MS) cells for medical treatments. iPS cells can be reprogrammed from regular cells and can become any cell type, including MS cells, which are important for tissue repair. However, a concern is that iPS cells might develop genetic changes that could affect their safety. Here researchers investigated these genetic changes during the creation and growth of iPS cells and their transformation into MS cells using advanced techniques such as chromosomal microarray and next-generation sequencing, alongside conventional methods. The study found that iPS cells often develop genetic alterations, which can persist as they are turned into MS cells. The results suggest that while iPS cells hold promise for regenerative medicine, careful monitoring of genetic stability is crucial. Future research should focus on improving methods to ensure safety of iPS cell-derived therapies.

This summary was initially drafted using artificial intelligence, then revised and fact-checked by the author.

## Introduction

Mesenchymal stromal/stem (MS) cells are a population of non-hematopoietic adherent cells derived from bone marrow, adipose tissue or placental tissue. They can differentiate into various types of tissues, such as bone, cartilage and adipose tissue^1^. MS cells have great potential for clinical applications, particularly in tissue repair and regeneration^2^. Recently, there has been a growing interest in generating functional MS cells from induced pluripotent stem (iPS) cells as a potential source of high-quality cells for regenerative medicine^3,4^.

iPS cell-derived MS cells (iMS cells) have been successfully generated and exhibit phenotypes and biological functions similar to MS cells. iMS cells are more homogeneous and predictable than primary MS cells, as they originate from a single iPS cell clone and their molecular signature remains stable among different batches^5^. iPS cells can provide stable and reliable sources of iMS cells, greatly expanding the clinical applications of iMS cells for treating various diseases, such as cardiovascular diseases and bone and cartilage disorders^3,5^.

Notably, iPS cells can experience genomic instability at any stage of their generation, and mutations may arise during differentiation to final cell products. This is a potential concern when using iMS cells for therapeutic applications^6^. Through replication, MS cells accumulate genomic instability, represented by abundant single-nucleotide variations (SNVs) and copy number alterations (CNAs) at late passages^7,8^.

Therefore, this study aimed to investigate the genomic changes occurring during the process of generating and passaging iPS cells and differentiating them into iMS cells, as well as the changes that occur during iMS cell passage. We utilized a range of techniques, including chromosome analysis, chromosomal microarray, short tandem duplication and next-generation sequencing (NGS), to comprehensively evaluate the genomic changes that occurred throughout the entire process. By defining the genomic scenarios occurring during iMS cell generation, differentiation and passaging, we hope to gain insights into the safety and efficacy of using iMS cells in regenerative medicine.

## Materials and methods

**Ethics**

All studies involving human and animal experiments were approved by the Institutional Review Board (P01-201910-31-005), the Institutional Animal Care and Use Committee of the Korea Research Institute of Bioscience and Biotechnology (KRIBB) (KRIBB-AEC-19153) and the Institutional Review Board of Seoul St. Mary’s Hospital (KC18BNSI0110).

### Generation of iPS cells from fibroblasts and characterization

#### Cell culture

Human skin fibroblasts (CRL-2097, American Type Culture Collection) were cultured in fibroblast medium containing 85% minimum essential medium-α (α-MEM, Thermo Fisher Scientific), supplemented with 15% FBS, 1% penicillin/streptomycin, 1× MEM non-essential amino acids (NEAAs) and 1% sodium pyruvate (all from Thermo Fisher Scientific). Primary umbilical cord-derived mesenchymal stem cells (UC-MS cells, human; PCS-500-010) were obtained from American Type Culture Collection.

#### Derivation of iPS cells

To establish the integration-free iPS cell lines, we reprogrammed human skin fibroblasts into iPS cells using Sendai virus vectors (CytoTune-iPS 2.0 Sendai Reprogramming kit (Thermo Fisher Scientific) encoding Oct4, Sox2, Klf4 and c-Myc or non-viral episomal plasmid vectors (Episomal iPS cell reprogramming vectors, Thermo Fisher Scientific) encoding Oct4, Sox2, Klf4, l-Myc, Lin28A and shp53 based on a previous protocol with a slight modification^9^. To generate Sendai virus vector-based iPS cells (SV-iPS cells), human fibroblasts were counted and plated at a density of 1 × 10^5^ cells in a six-well plate. After 2 days, cells were transduced with SVs in fibroblast medium with 4 μg/ml of polybrene (Sigma) and incubated overnight at 37 °C in a CO_2_ incubator. The following day, the medium was replaced with fresh medium and incubated for 4 days, with the medium changed every other day. From day 6 onward, the medium was changed to iPS cell medium (mTeSR1; StemCell Technologies) and replaced daily until iPS cell colonies appeared. To generate episomal vector-based iPS cells (Epi-iPS cells), human fibroblasts (1 × 10^6^ cells per reaction) were electroporated with episomal vectors using the Neon Transfection System (Thermo Fisher Scientific). The electroporated cells were plated in a well of a six-well plate and incubated for 5 days in fibroblast medium, with the medium changed every other day. Cells were plated at a density of 1–5 × 10^4^ cells per well in a six-well plate. The following day, the medium was replaced with iPS cell medium mTeSR1 every day until iPS cell colonies appeared. iPS cell colonies were manually selected, transferred to 12-well plates and expanded in mTeSR1 with daily changes for additional analysis. The iPS cell colonies were manually passaged every 5–7 days throughout the experiments.

#### ALP staining

Alkaline phosphatase (ALP) staining was performed for 30 min at room temperature using the Vector Red Alkaline Phosphatase Substrate kit I (Sigma) according to the manufacturer’s instructions. Images were captured using a microscope (IX51; Olympus).

#### Immunocytochemistry staining

For immunocytochemical staining, iPS cells were fixed in 4% formaldehyde in phosphate-buffered saline (PBS) for 15 min and permeabilized with 0.1% Triton X-100 in PBS for 30 min at room temperature. After blocking with 4% bovine serum albumin for 2 h, the fixed cells were incubated with primary antibodies overnight at 4 °C. The following primary antibodies were used: Oct3/4 (H-134) (1:100, sc-9081, Santa Cruz), Nanog (1:40, AF1997, R&D Systems), Tra-1-81 (1:100, MAB4381, Millipore), SSEA-3 (1:30, MAB1434, R&D Systems), Tra-1-60 (1:100, MAB4360, Millipore), SSEA-4 (1:30, MAB1435, R&D Systems), Tuj1 (1:500, 802001, Biolegend), Nestin (1:100, MAB5326, Millipore), Desmin (1:50, AB907, Millipore), Actin, alpha-Smooth Muscle (α-SMA) (1:200, A5228, Sigma), HNF3beta/FOXA2 (1:100, 07-633, Millipore), SOX17 (1:50, MAB1924, R&D Systems) and phospho-Histone H2A.X (Ser139), clone JBW301 (1:500, 05-636, Millipore). Primary antibodies were detected using Alexa Fluor 594 and 488 (1:200; Thermo Fisher Scientific) conjugated with secondary antibodies. Nuclei were stained with 4,6-diamidino-2-phenylindole (DAPI). All fluorescence images were acquired using an Axio VertA.1 microscope (Carl Zeiss) and a LSM 800 Laser Scanning Confocal Microscope (Carl Zeiss). The antibodies used in this study are listed in Supplementary Table 1.

#### Three-germ layer differentiation of iPS cells

To validate the spontaneous three-germ layer differentiation potential of iPS cells, in vitro differentiation was induced by the formation of embryonic bodies (EBs), as previously described^9^. For EB formation, clumps of iPS cells were cultured in EB medium consisting of knockout DMEM supplemented with 10% KSR, 1% NEAA, 0.1 mM β-mercaptoethanol and 1 mM l-glutamine for 5 days on Petri dishes. The medium was changed every other day. Floating EBs were seeded on gelatin-coated plates and cultured for an additional 10 days. The medium was changed every other day.

#### Teratoma formation

For teratoma formation, iPS cells (2 × 10^6^ cells) were prepared in 50 μl of PBS/Matrigel (BD Biosciences) and injected subcutaneously into the dorsal flank of 4-week-old CAnN.Cg-Foxn1 nu/CrljOri mice (ORIENT Bio. Inc.). Ten weeks after injection, the teratomas were collected, dissected and fixed in 4% formaldehyde. The teratoma tissues were then processed, embedded in paraffin wax and sectioned at 5 μm. Sectioned teratomas were analyzed using hematoxylin and eosin staining.

#### Mycoplasma test

The absence of mycoplasma contamination was assessed via PCR using the EZ-PCR Mycoplasma detection kit (Biological Industries).

### Differentiation of iPS cells into iMS cells

#### Differentiation into iMS cells

SV-iPS cells and Epi-iPS cells were differentiated into MS cells (SV-iMS cells and Epi-iMS cells, respectively) using the STEMdiff Mesenchymal Progenitor kit (StemCell Technologies) according to the manufacturer’s instructions. SV-iPS cells and Epi-iPS cells were plated at a density of 5 × 10^4^ cells/cm^2^ on six-well plates precoated with Matrigel and cultured in mTeSR1 for 2 days with daily medium changes. After washing with Dulbecco’s phosphate-buffered saline (DPBS), iPS cells were cultured with STEMdiff-ACF mesenchymal induction medium for 4 days, with daily medium changes. After washing with DPBS (Thermo Fisher Scientific), iPS cells were cultured with MesenCult-ACF medium for 2 days with daily medium changes and then passaged into six-well plates precoated with MesenCult-ACF Attachment Substrate. Cells were subcultured with 0.25% (w/v) trypsin–EDTA until they reached approximately 70–80% confluency. The medium was changed daily for 15 days. Finally, differentiated iMS cells were cultured and expanded at a density of 1 × 10^4^ cells/cm^2^ in MesenCult-ACF Medium or MS cell medium containing 90% α-MEM, supplemented with 10% FBS, 1% penicillin/streptomycin, 1× MEM NEAA, 1× GlutaMAX and 5 ng/ml basic fibroblast growth factor (R&D Systems).

#### Flow cytometry

Single-cell dissociated SV-iMS cells and Epi-iMS cells were stained with PE-conjugated CD73, PE-conjugated CD105, PE-conjugated CD90 (Miltenyi Biotec); CD34/CD45 Cocktail, FITC, PE (Thermo Fisher Scientific); FITC-conjugated HLA-ABC and FITC-conjugated HLA-DR (BioLegend) for 30 min and protected from light. The stained cells were washed twice with PBS. Flow cytometric analysis was performed using a BD Accuri C6 flow cytometer (BD Biosciences) to detect the cell surface markers of SV-iMS cells and Epi-iMS cells. The antibodies used in this study are listed in Supplementary Table 1.

#### Tri-lineage differentiation of iMS cells

Tri-lineage differentiation of iMS cells was performed using an Osteogenesis, Adipogenesis and Chondrogenesis Differentiation kit (Thermo Fisher Scientific), according to the manufacturer’s protocol. iMS cells were seeded at a density of 5000 cells/cm^2^ in 24-well plates and incubated in the MS cell medium. The next day, the cells were transferred to osteogenic, adipogenic and chondrogenic differentiation medium and incubated until differentiation was complete. Fresh differentiation medium was changed every 3 days. After differentiation, Alizarin Red, Oil Red O and Alcian blue dyes (Sigma) were used to visualize the presence of calcified matrix, lipid droplets and cartilage matrix, respectively. Briefly, after fixing the differentiated cells with 4% formaldehyde for 10 min, the cells were washed three times with distilled water and stained with Alizarin Red (2%), Oil Red O (0.5%) and Alcian Blue solution (1%) at room temperature.

#### Assessment of tumorigenicity of iMS cells

To study the tumorigenic properties of iMS cells, we used ten NSG mice (NOD scid gamma mice; NOD.Cg-Prkdc^scid^Il2rg^tm1Wjl^/SzJ, Jackson Laboratory), comprising five females and five males, all 7 weeks old. Each mouse was injected subcutaneously with a cell suspension of 1.0 × 10^7^ iMS cells combined with Matrixgel (BD Biosciences) in a volume of 200 μl. We monitored the iMS cell implantation site for morphological changes for up to 12 months and meticulously recorded our observations. At the end of the observation period, we performed thorough necropsies on each animal. The retrieved tissues were immediately fixed in formalin and prepared for detailed pathological examination.

### RT–qPCR)

Total RNA was isolated using the RNeasy Mini kit (Qiagen) according to the manufacturer’s protocol. RNA (2 μg) was reverse transcribed using a SuperScript VILO cDNA Synthesis Kit (Thermo Fisher Scientific). Quantitative PCR with reverse transcription (RT–qPCR) was performed using the SYBR Green Real-time PCR Master Mix (Roche Diagnostics GmbH) on a 7500 Fast Real-Time PCR System (Applied Biosystems). The expression of tissue-specific genes were measured and normalized to that of the housekeeping gene *GAPDH*. The primers used are listed in Supplementary Table 2.

### Evaluation of genomic stability

#### Karyotyping

Chromosomal analyses of iPS cell lines were performed according to a previously described method^10^. Briefly, cultured cells were treated by colcemid. Cells were collected using trypsin and treated by prewarmed hypotonic KCl solution. After fixation with a 1:3 acetic acid:methanol solution, slides were prepared for chromosomal analysis using the trypsin–Giemsa banding technique. At least 20 metaphase samples were analyzed.

#### STR analysis

Genomic DNA was extracted using the Wizard Genomic DNA Purification kit (Promega) and used for further genomic stability analyses, including short tandem repeat (STR) analysis. STR analysis was performed using AmpFlSTR Identifier PCR amplification to confirm iPS cell line identity (Applied Biosystems), according to the method used in previous study^11^.

#### Chromosomal microarray

CNAs were analyzed using SurePrint G3 Human CGH+SNP microarray 4× 180K kit (Agilent Technologies) and/or the Affymetrix CytoScan Dx Assay kit (Affymetrix), according to the manufacturers’ recommended methods used in our previous studies^8^. Identified CNAs were validated using an alternative platform (either SurePrint or Affymetrix, as applicable). Genomic Workbench 7.0.4.0 software (Agilent Technologies) and Chromosome Analysis Suite Dx (ChAS Dx) v1.3 Affymetrix Software package were used to determine CNAs.

#### NGS

We used the Oncomine Comprehensive Panel v3M (Thermo Fisher Scientific,) and Oncomine Childhood Cancer Research Assay kit (Thermo Fisher Scientific) according to the manufacturer’s instruction^12^. Sequencing was performed on an Ion S5 XL Sequencer (Thermo Fisher Scientific) and analyzed using the Ion Torrent Server software (Thermo Fisher Scientific). Detected mutations were validated using the TruSight Oncology 500 assay (Illumina). DNA libraries were prepared, enriched, and sequenced on NextSeq 550 Dx (Illumina). Sequencing data were analyzed using the TruSight Oncology 500 Local App Version 2.2 (Illumina). Elaborate sequence data in FASTQ format were adjusted and annotated according to the hg19 human reference genome. The variant allele fraction was calculated by dividing the number of mutant sequencing reads by the total number of reads. All detected mutations were verified manually using Integrative Genomic Viewer19.

### RNA sequencing

To evaluate gene expression changes between reprogramming methods, total RNA sequencing was performed on representative cells (SV-iPS cell and Epi-iPS cell) at both mid- and late passages. Libraries were prepared using the Illumina Stranded Total RNA Prep with Ribo-Zero Plus (Illumina) with 800 ng of total RNA, followed by sequencing on an Illumina NovaSeq-6000 sequencer (Illumina). The sequencing data were analyzed using the CLC Genomics Workbench 20.0.4 (Qiagen). The FASTQ files were processed and annotated using the hg38 human reference genome. Gene expression quantification was normalized to transcripts per million, and fold changes in differentially expression genes (DEGs) were calculated.

The chromosomal instability 25 (CIN25) gene signature, which includes specific genes whose expression is consistently correlated with total functional aneuploidy^13^, was evaluated by calculating the log_2_ fold change in expression levels. Statistical significance for each DEG was determined using a *P* value of <0.05. To further explore the impact of passaging on gene expression, we conducted Gene Set Enrichment Analysis (GSEA) to identify changes in DEGs. A false discovery rate *q* value of <0.05 and family-wise error rate *P* value of <0.05 was considered statistically significant.

### Statistical analysis

The data are presented as the means ± s.d. of at least three separate experiments. Comparisons between two groups were analyzed using Student’s *t*-tests. Significance was established at *P < 0.05 and **P < 0.01. All analyses were performed using SPSS for Windows (version 24.0; IBM Corp.).

## Results

### Characterization of integration-free iPS cells

**Fig. 1:**
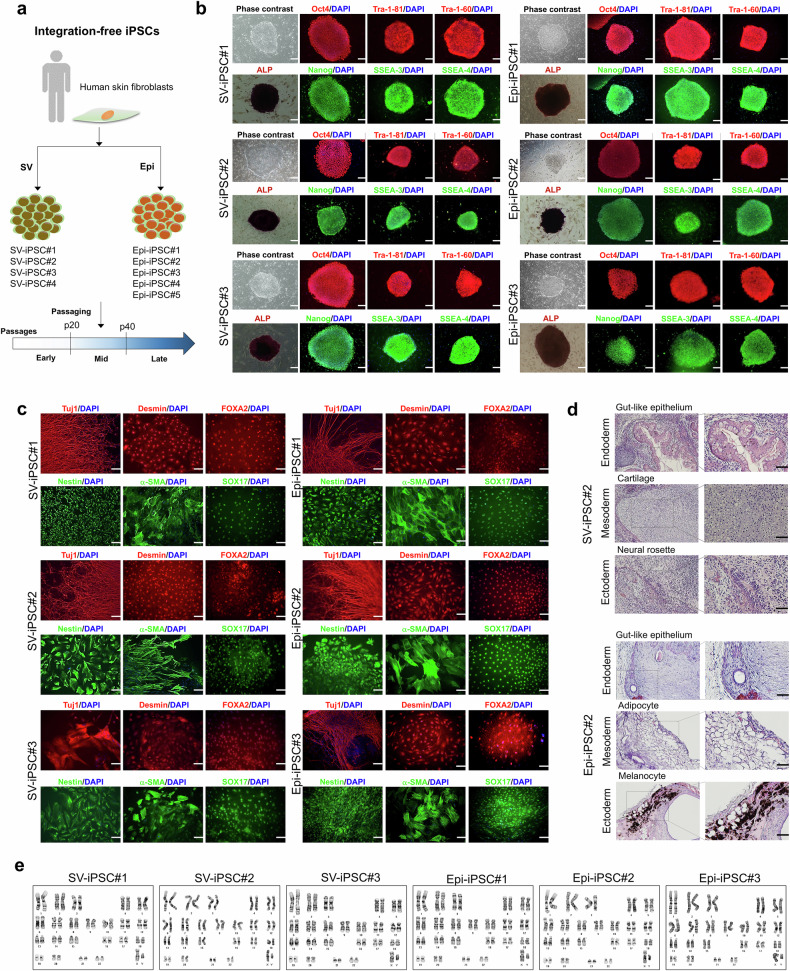
Generation and characterization of iPS cells. **a**, An overview of integration-free iPS cell generation. Pluripotency characterization was performed using passages 7–10. **b**, Immunocytochemistry analysis of pluripotency markers (Oct4, Nanog, Tra-1-81, Tra-1-60, SSEA-3 and SSEA-4) and ALP staining in iPS cells generated using SV (SV-iPS cell) and episomal vectors (Epi-iPS cell). Nuclei were stained by DAPI. Scale bar, 200 μm. **c**, Immunocytochemistry analysis of markers for the three-germ layers (Tuj1 and Nestin (ectoderm); FOXA2 and SOX17 (endoderm); Desmin and α-SMA (mesoderm)) in in vitro differentiated SV-iPS cells and Epi-iPS cells. Scale bar, 100 μm. **d**, Hematoxylin and eosin staining of teratomas generated with SV-iPS cells and Epi-iPS cells. Differentiation into multiple derivatives of three-germ layers is shown: ectoderm (melanocyte, neural rosette), endoderm (gut-like epithelium) and mesoderm (adipocyte, cartilage). Scale bar, 50 μm. **e**, Normal karyotype of Epi-iPS cells and SV-iPS cells.

We generated four independent SV-iPS cells and five Epi-iPS cells for use in this study (Fig. 1a). Fully reprogrammed iPS cell lines exhibited typical human embryonic stem cell-like morphology (Fig. 1b). Pluripotency was confirmed by classical assays. Immunocytochemical analysis showed strong expression of the transcription factors Oct3/4 and Nanog, as well as the surface markers SSEA-3, SSEA-4, TRA-1-60 and TRA-1-81 in undifferentiated SV-iPS cells and Epi-iPS cells (Fig. 1b). In addition, the analysis of in vitro differentiation potential of SV-iPS cells and Epi-iPS cells indicated their contribution to all three germ layers (ecto-, meso- and endoderm) (Fig. 1c and Supplementary Fig. 2c). The potential of SV-iPS cells and Epi-iPS cells to differentiate into the three germ layers was further verified in vivo using teratoma assays (Fig. 1d). STR analysis confirmed the genetic identity of all SV-iPS cell and Epi-iPS cell lines with the parental cells (Supplementary Fig. 1a). SV-iPS cells and Epi-iPS cells retained their normal karyotype for over 60 passages (Fig. 1e) without mycoplasma contamination (Supplementary Fig. 1b). The pluripotency of SV-iPS cells and Epi-iPS cells used for the genomic stability and tumorigenicity studies was confirmed by the presence of pluripotency markers (Supplementary Fig. 2a,b).

### Characterization of iMS cells

**Fig. 2:**
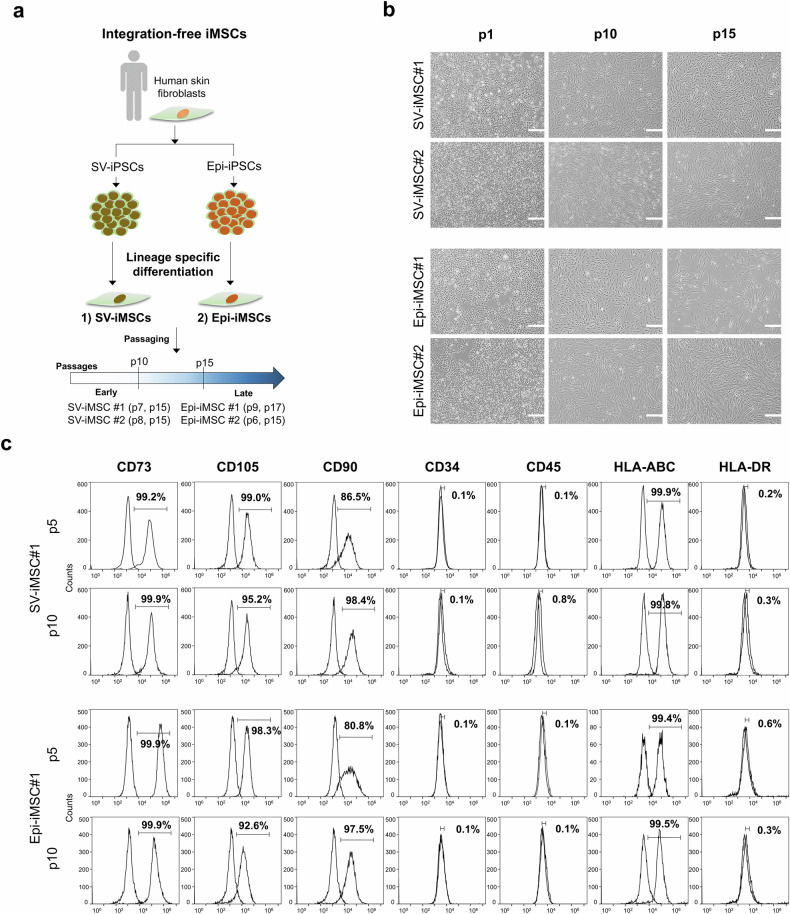
Generation and characterization of iMS cells. **a**, An overview of integration-free iMS cell generation. MS cell characterization was performed using passages 4–6. **b**, Phase-contrast images of iMS cells generated using SV (SV-iMS cell) and episomal vectors (Epi-iMS cell). Scale bar, 200 μm. **c**, Flow cytometric analysis of MS cell markers (CD73, CD105 and CD90), hematopoietic markers (CD34 and CD45), HLA class I (HLA-ABC) and HLA class II (HLA-DR) in passaged SV-iMS cell 1 (at p5 and p10) and Epi-iMS cell 1 (at p5 and p10).

**Fig. 3:**
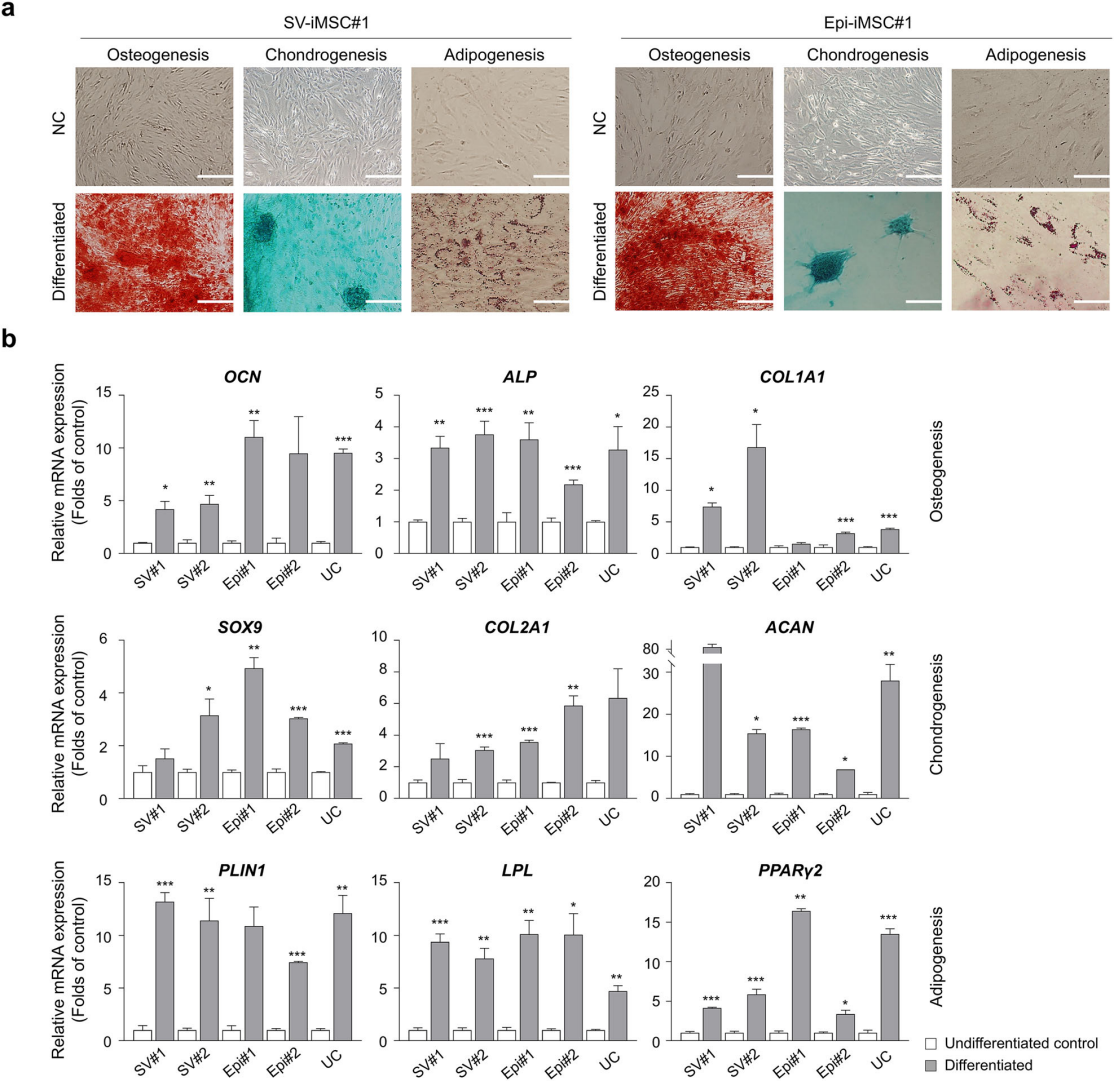
Differentiation potential of iMS cells using two integration-free methods: SV-iPS cells and Epi-iPS cell into osteoblasts, chondrocytes and adipocytes in vitro. **a**, Representative staining of osteoblasts (Alizarin red staining, left, scale bar, 200 μm), chondrocytes (Alcian blue staining, middle, scale bar, 100 μm), adipocytes (Oil red O staining, right, scale bar, 50 μm) and negative control (NC) groups. **b**, Relative expression levels of lineage-specific markers measured by RT–qPCR: *OCN*, *ALP* and *COL1A1* for osteoblasts; *SOX9*, *COL2A1* and *ACAN* for chondrocytes; and *PLIN1*, *LPL* and *PPARγ2* for adipocytes. The UC-MS cells were verified as the control primary MS cells. Data represent means ± s.d. of three independent experiments. *P < 0.05, **P < 0.01 and ***P < 0.001 compared with undifferentiated control (*t*-test). SV#1, SV-iMS cell 1; SV#2, SV-iMS cell 2; Epi#1, Epi-iMS cell 1; Epi#2, Epi-iMS cell 2; UC, UC-MS cell.

SV-iPS cells and Epi-iPS cells reprogrammed from human fibroblasts were subjected to mesenchymal differentiation to produce iMS cells for 21 days in a commercial MS cell differentiation medium (Fig. 2a). Our results revealed that both SV-iMS cells and Epi-iMS cells exhibited a typical fibroblast-like morphology (Fig. 2b). During passage, the cells constituted a homogeneous population of fibroblasts (Fig. 2b). The expression of MS cell-specific cell surface markers in passaged iMS cells was evaluated using flow cytometry. Flow cytometric analysis showed that SV-iMS cell 1 (at p5 and p10) and Epi-iMS cell 1 (at p5 and p10) were negative for antigens CD34, CD45 and HLA-DR and positive for known antigens of MS cells, CD73, CD90, CD105 and HLA-ABC^14–17^. Less than 5% of the analyzed cell population expressed CD34 or CD45 at any passage. The expression levels of CD73, CD105, CD90 and HLA-ABC were stable and high throughout all passages (Fig. 2c). We observed that MS cell-specific surface marker properties were preserved after passaging for p15–17, as confirmed via RT–qPCR analysis (Supplementary Fig. 3a). MS cell characteristics of SV-iMS cells and Epi-iMS cells used for genomic stability and tumorigenicity studies were confirmed by the presence of MS cell-specific markers and/or absence of pluripotency marker (Supplementary Fig. 3b). The typical MS cell-like properties of SV-iMS cells and Epi-iMS cells were assessed based on their multilineage differentiation potential into osteoblasts, chondrocytes and adipocytes. In osteogenic differentiation, both SV-iMS cells and Epi-iMS cells formed extracellular calcium matrices, as demonstrated by Alizarin Red S staining (Fig. 3a). Chondrogenic differentiation was confirmed by the accumulation of sulfated proteoglycans, visualized by Alcian blue staining (Fig. 3a). For adipogenic differentiation, the accumulation of lipid droplets within the cells was observed through Oil Red O staining (Fig. 3a). Real-time PCR analysis further supported these findings by detecting the expression of lineage-specific markers: alkaline phosphatase (*ALP*), osteocalcin (*OCN*) and collagen type I (*COL1A1*) for osteoblasts; *SOX9*, collagen type II (*COL2A1*) and aggrecan (*ACAN*) for chondrocytes; and perilipin1 (*PLIN1*), lipoprotein lipase (*LPL*) and peroxisome proliferator-activated receptor-gamma (*PPARγ2*) for adipocytes (Fig. 3b). No substantial differences were observed in the differentiation capacity between SV-iMS cells, Epi-iMS cells and control UC-MS cells, indicating comparable lineage-specific differentiation potential across all groups.

The in vivo tumorigenicity assay demonstrated that all NSG mice survived for 12 months after receiving an injection of an iMS cell suspension. Throughout the observation period, no tumors were detected at the injection sites. Additionally, tissue specimens taken from the injection sites showed no microscopic pathological abnormalities.

### Replication stress and DNA damage

Elevated levels of phosphorylation of histone H2AX on serine 139 (γH2AX) in iPS cells indicate replication stress and DNA damage, both of which are crucial to monitor for genomic stability^18^. We examined and compared the level of γH2AX in the human skin fibroblasts (p6 versus p30), SV-iPS cells (p11 versus p41), Epi-iPS cells (p35 versus p42), SV-iMS cells (p7 versus p15) and Epi-iMS cells (p9 versus p17). In a control experiment, a significant increase in γH2AX foci staining was observed when the fibroblasts (p6) were treated with doxorubicin and aphidicolin, known inducers of replication stress (Supplementary Fig. 4a,b). Both SV-iPS cells and Epi-iPS cells exhibited higher basal levels of γH2AX compared with fibroblasts and iMS cells, indicating greater replication stress and DNA damage. As expected, replication stress was more pronounced in late-passage fibroblasts, SV-iPS cells, Epi-iPS cells, SV-iMS cells and Epi-iMS cells compared with early passage cells, leading to increased genomic instability and associated aberrations. Notably, γH2AX levels were not significantly affected by different reprogramming methods such as non-integrating SV and Epi methods (Supplementary Fig. 4c,d), suggesting that the replication stress levels were similar in iPS cells regardless of the reprogramming method used.

### Genomic instability

**Fig. 4:**
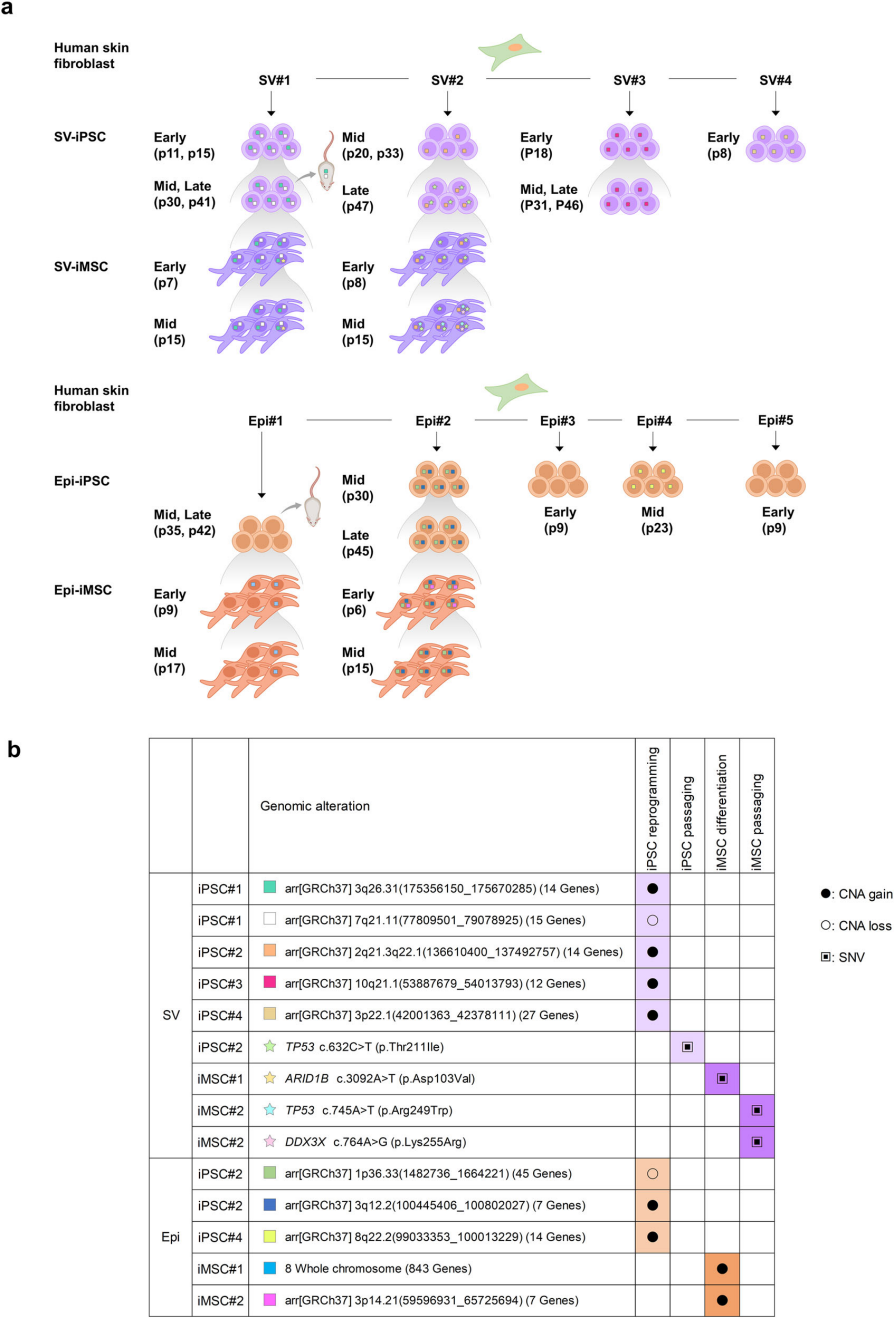
The genomic alterations in iPS cells and iMS cells through reprogramming, differentiation and passaging. **a**, Nine iPS cells were produced using two integration-free methods: SV (top, SV-iPS cell) and episomal vectors (bottom, Epi-iPS cell). Two iMS cell lines differentiated from each iPS cell lines are depicted, including their further passages. In vivo genomic stability was assessed using teratomas, symbolized by a mouse. **b**, Reprogramming-induced CNAs were found in all SV-iPS cells and two Epi-iPS cells. All differentiated iMS cell lines were found to carry exhibited CNA or SNVs. Notably, the all detected genomic aberrations were distinct and showed no recurring pattern. Each alteration was validated using independent methodologies.

Genomic stability was evaluated in four SV-iPS cells and five Epi-iPS cells during processing. Additionally, two iMS cell lines differentiated from each of the two iPS cell lines were included along with their subsequent passages. Of the nine iPS cell lines, six harbored aberrant clones carrying one or two CNAs that were not detected in fibroblasts. All four SV-iPS cells and two of the five Epi-iPS cells exhibited CNAs, which occurred immediately after reprogramming and persisted during subsequent passaging, teratoma formation and iMS cell differentiation (Fig. 4a). Consequently, all four cell lines that differentiated into iMS cells acquired additional CNAs or SNVs. SNVs have only been developed in cell lines derived from SV-iPS cells. All CNAs (Supplementary Table 3) and SNVs were unique and non-recurrent (Fig. 4b).

In SV-iPS cell 1, two CNAs developed after reprogramming and were present in iPS cell passaging (p11–41), early and late-passage iMS cells, and teratoma tissues. The first CNA was 1.27 MB loss in chromosome 7q21.11, with a CNA fraction of 0.85–0.98, and the second CNA was a 0.31 MB gain in chromosome 3q26.31, with a CNA fraction of 0.52–0.72. Additionally, a novel SNV in the *ARID1B* gene, identified as Q1031L, emerged in a very small fraction (0.53%) during differentiation. This variant became enriched, reaching 4% in late passages (p15), and was predicted to impact splicing. SV-iPS cell 2 harbored a 0.88-MB-sized gain in chromosome 2q21.3–22.1, which was retained throughout the passages (p20–47) and differentiated into iMS cells. In addition, *TP53* mutation (T211I) appeared in the late-passage iPS cell (p47) and persisted during iMS cell differentiation with a significant allelic fraction of 0.33–0.47. Furthermore, two additional SNVs were found in *TP53* (R249W) and *DDX3X* (K255R) cells in the later passage iMS cell (p15), with small allelic fractions of 0.14 and 0.27, respectively. SV-iPS cell 3 exhibited reprogramming-induced CNAs, including a 0.13 MB gain on chromosome 10q21.1, whereas SV-iPS cell 4 showed a 0.38 MB gain on chromosome 3p22.1.

Epi-iPS cell 1 cells did not exhibit any genomic aberrations during reprogramming and passaging (p35–42) as well in teratoma tissues. After iMS cell differentiation, trisomy 8 emerged and persisted until late-passage p17. Epi-iPS cell 2 harbored two CNAs that remained throughout passaging (p30–45) and differentiated into iMS cells (p6–15). The first CNA was 0.18 MB loss in chromosome 1p36.3, with a CNA fraction of 0.81–0.92, and the second CNA was a 0.36 MB gain in chromosome 3q12.2, with a CNA fraction of 0.45–0.60. In addition, a 0.9 MB gain in 3p14.21 was acquired in the late-passage iPS cell (p45) with a significant burden (0.40–0.50), and this was retained in the iMS cells. Epi-iPS cell 4 exhibited a 1 MB gain on chromosome 8q22.2 with a CNA fraction of 0.61 at a moderate passage (p23), whereas Epi-iPS cells 3 and 5 did not show any genomic aberrations.

In the analysis of the original fibroblast line CRL-2097 across passages 5, 15 and 30, no significant CNAs or SNVs were observed, confirming the impact of reprogramming and differentiation on genomic stability (data not shown).

### Changes in gene expression by iPS cell reprogramming methods

The expression levels of 25 genes in the CIN25 signature were evaluated in both mid- and late-passage iPS cells. As presented in Supplementary Table 4, SV-iPS cells (p20 versus p41) exhibited significantly upregulated expression in 24 out of 25 genes, while Epi-iPS cells (p35 versus p42) showed upregulation in 2 out of 25 genes. Downregulated expression was observed in 1 out of 25 genes for SV-iPS cells and in 7 out of 25 genes for Epi-iPS cells. GSEA analysis was conducted to investigate differences in signaling hallmark and Kyoto Encyclopedia of Genes and Genomes pathways (Supplementary Table 5). Supplementary Fig. 5 highlights significantly enriched pathways, including MYC targets, DNA repair, G2M checkpoint, p53 and cancer-related pathways in SV-iPS cells, while Epi-iPS cells showed enrichment in a few pathways including hypoxia and glycolysis.

## Discussion

Genetic instability is a significant concern in cell therapy as it is linked to cellular function and/or tumorigenesis. In MS cells, genetic instability typically results in cellular senescence, which is associated with a decrease in the therapeutic efficacy of the cells^8,19^. However, in iPS cells, various genomic aberrations, including CNAs, SNVs and structural variations, have been observed^20,21^. Evidence suggests that enhanced cell culture not only leads to prolonged expression of transcription factors, such as c-Myc, but also to the inactivation of *TP53*, and these factors may play a significant role in tumorigenesis. Although it remains unclear whether these genomic aberrations are actual risk factors for adverse events, their presence of genomic aberrations has raised safety concerns^22^. iPS cells have become increasingly attractive for regenerative medical applications. However, concerns about their genomic instability and carcinogenic potential have limited their clinical use. To address these concerns, recent advancements in iPS cell technology have employed several integration-free methods^23,24^. These methods aim to minimize the risk of genomic instability and carcinogenesis associated with iPS cell use^25^.

In this study, we successfully generated iPS cells using two integration-free methods: SV and episomal vectors. Subsequently, we induced differentiation of these cells into iMS cells. We confirmed the principal characteristics of both iPS cells and iMS cells, maintaining genetic parentality using STR analysis. However, a comprehensive genomic evaluation revealed various genomic aberrations.

γH2AX staining indicated higher replication stress and DNA damage in iPS cells, regardless of the reprogramming method used. Notably, all SV-iPS cell lines exhibited CNAs during the reprogramming process, whereas only 40% of Epi-iPS cells showed such alterations. Additionally, SNVs were observed in SV-iPS cells and SV-iMS cells, but not in Epi-iPS cell and Epi-MS cell lines. Gene expression analysis revealed that the majority of genes in the CIN25 signature were upregulated in late-passage SV-iPS cells compared with mid passage, suggesting increased chromosomal instability with prolonged passaging. In contrast, Epi-iPS cells showed upregulation in only 2 out of 25 genes, indicating a more stable gene expression profile. Similarly, GSEA revealed that in SV-iPS cells, significant pathways related to DNA repair, p53 and cancer were enriched during passaging, while these changes were less pronounced in Epi-iPS cells. This further supports the notion that Epi-iPS cells maintain better genomic stability than SV-iPS cells. Several studies have reported small genomic alterations in iPS cell lines, including CNAs and SNVs, which may reflect the mutagenic effects of the reprogramming process itself^26–28^. Studies have demonstrated that different reprogramming methods, whether integrating or non-integrating, have varied impacts on replication stress and genomic integrity^29,30^. Non-integrating methods, including SV-, Epi-, mRNA- and protein-based methods, mitigate the risks associated with direct genetic modification, making them safer for clinical applications and research^30–32^. However, little is known about which of these non-integrating methods is superior in preserving genomic stability. Our findings highlight the relative stability of the Epi-based method. Further comparative studies between iPS cells generated by various non-integrating and integrating reprogramming methods are crucial for understanding and mitigating risks, thereby promoting safer and more effective clinical applications.

The detected genomic aberrations were individualized; therefore, we could not identify common changes. Analytical technologies, SNP arrays and NGS with sufficient depth are required to identify genomic aberrations^33,34^. Most genetic aberrations were interstitial duplications detected by the SNP array, which were primarily acquired during the reprogramming process. These amplified regions included genes with critical functions: *MCM6* and *PTPRG* in cellular replication; *DARS, RPL30* and *POP1* in protein synthesis; and *HRPS12* and *STK3* in maintaining cellular homeostasis. We also observed interstitial losses in *CDK11A*, *CDK11B* and *SSU72* genes, which encode enzymes vital for cell division. During iMS cell differentiation, a clone with trisomy 8 developed, leading to the amplification of the *MYC* oncogene^35–37^. This clone rapidly supplanted half of the iMS cell population, indicating high replicative capacity. SNVs were detected in *ARID1B*, *TP53* and *DDX3X* genes via NGS. The *ARID1B* gene functions to regulate gene expression through chromatin remodeling and is associated with several developmental health conditions and cancers^38–40^. The *DDX3X* gene encodes a DEAD-box RNA helicase that plays a crucial role in cell cycling, apoptosis and tumorigenesis. Notably, two *TP53* mutations were observed, making it the only well-known tumor suppressor gene for which mutations have been detected in multiple iPS cell lines^41^. The late-occurring *TP53* mutation is a well-defined oncogenic variant (COSM43629) that is accompanied by a *DDX3X* mutation, indicating a sudden increase in genomic instability.

This study had several limitations. First, the reprogramming process was conducted using a single set of purchased human fibroblasts, which limited our ability to analyze potential genomic aberrations across a broader range of donor-specific differences. Second, we did not confirm the functional consequences of the detected genetic aberrations. Third, our data revealed a high incidence of genomic aberrations, especially SNVs, in cell lines generated using the SV method. However, further verification through additional studies is required.

Our study suggests that iPS cells are prone to genomic aberrations throughout all processes, including differentiation into iMS cells. iPS cells generated using the SV method exhibit greater genomic instability compared with those generated with episomal vectors. SV-iPS cells displayed a higher frequency of CNAs and SNVs, along with increased expression of chromosomal instability genes, indicating a higher risk of genomic aberrations during extended culture. Notably, CNAs are frequently associated with replication-related genes. The identification of *TP53* mutations underscores the vulnerability of the gene and highlights the need for careful scrutiny when preparing iPS cells and derived cell lines.

## Supplementary information

Supplementary Information
