# Supplementary material for: Tracing genomic instability in induced mesenchymal stromal cell manufacture: an integration-free transfection approach
Source: Exp Mol Med. 2025 Apr 14;57(4):900–9. doi: 10.1038/s12276-025-01439-8 (PMC12046023; doi:10.1038/s12276-025-01439-8)
Supplement: Supplementary file 1 — Supplementary Information [file 12276_2025_1439_MOESM1_ESM.pdf]

Supplementary Material

Tracing Genomic Instability in Induced Mesenchymal Stromal Cell Manufacture: An Integration-Free Transfection Approach

Jong-Mi Lee[1,2,*], Chae Yeon Lee[1,3,*], Binna Seol[4,*] , Chan Kwon Jung[5], Yonggoo Kim[1,2], Dain Kang[1], Haein Yu[1], Yuna Hong[1,3], Cho Lok Song[4], Yee Sook Cho[4,6,¶], Myungshin Kim[1,3,¶]

*These authors contributed equally to this work

[1]Catholic Genetic Laboratory Center, Seoul St. Mary's Hospital, College of Medicine, The Catholic University of Korea, Seoul, Republic of Korea
[2]Department of Laboratory Medicine, College of Medicine, The Catholic University of Korea, Seoul, Republic of Korea
[3]Department of Medical Sciences, Graduate School of The Catholic University of Korea, Seoul, Republic of Korea
[4]Stem Cell Research Laboratory, Immunotherapy Research Center (IRC), Korea Research Institute of Bioscience and Biotechnology (KRIBB), 125 Gwahak-ro, Yuseong-gu, Daejeon 34141, Republic of Korea
[5]Department of Hospital Pathology, College of Medicine, The Catholic University of Korea, Seoul, Republic of Korea
[6]Department of Bioscience, KRIBB School, University of Science & Technology, 113 Gwahak-ro, Yuseong-gu, Daejeon 34113, Republic of Korea

¶Corresponding authors

The file contains

Supplementary Figures 1 to 5

Supplementary Tables 1 to 5

a

Locus/Clone	Fibroblasts		SV-iPSC#1		SV-iPSC#2		SV-iPSC#3		Epi-iPSC#1		Epi-iPSC#2		Epi-iPSC#3	
D8S1179	12	12	12	12	12	12	12	12	12	12	12	12	12	12
D21S11	29	31.2	29	31.2	29	31.2	29	31.2	29	31.2	29	31.2	29	31.2
D7S820	12	12	12	12	12	12	12	12	12	12	12	12	12	12
CSF1PO	12	13	12	13	12	13	12	13	12	13	12	13	12	13
D3S1358	15	15	15	15	15	15	15	15	15	15	15	15	15	15
TH01	6	9.3	6	9.3	6	9.3	6	9.3	6	9.3	6	9.3	6	9.3
D13S317	11	12	11	12	11	12	11	12	11	12	11	12	11	12
D16S539	9	11	9	11	9	11	9	11	9	11	9	11	9	11
D2S1338	20	23	20	23	20	23	20	23	20	23	20	23	20	23
D19S433	13	15	13	15	13	15	13	15	13	15	13	15	13	15
vWA	17	18	17	18	17	18	17	18	17	18	17	18	17	18
TPOX	10	11	10	11	10	11	10	11	10	11	10	11	10	11
D18S51	13	18	13	18	13	18	13	18	13	18	13	18	13	18
D5S818	11	12	11	12	11	12	11	12	11	12	11	12	11	12
FGA	24	26	24	26	24	26	24	26	24	26	24	26	24	26
**Gender**	XY		XY		XY		XY		XY		XY		XY	

b

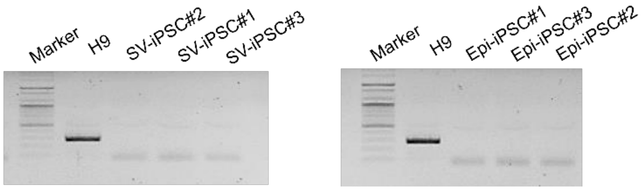

Supplementary Figure 1. (a) Short tandem repeat (STR) analysis of human skin fibroblasts, induced pluripotent stem cells (iPSCs) generated using Sendai virus (SV-iPSC) and episomal vectors (Epi-iPSC). (b) Mycoplasma tests of SV-iPSC and Epi-iPSC.

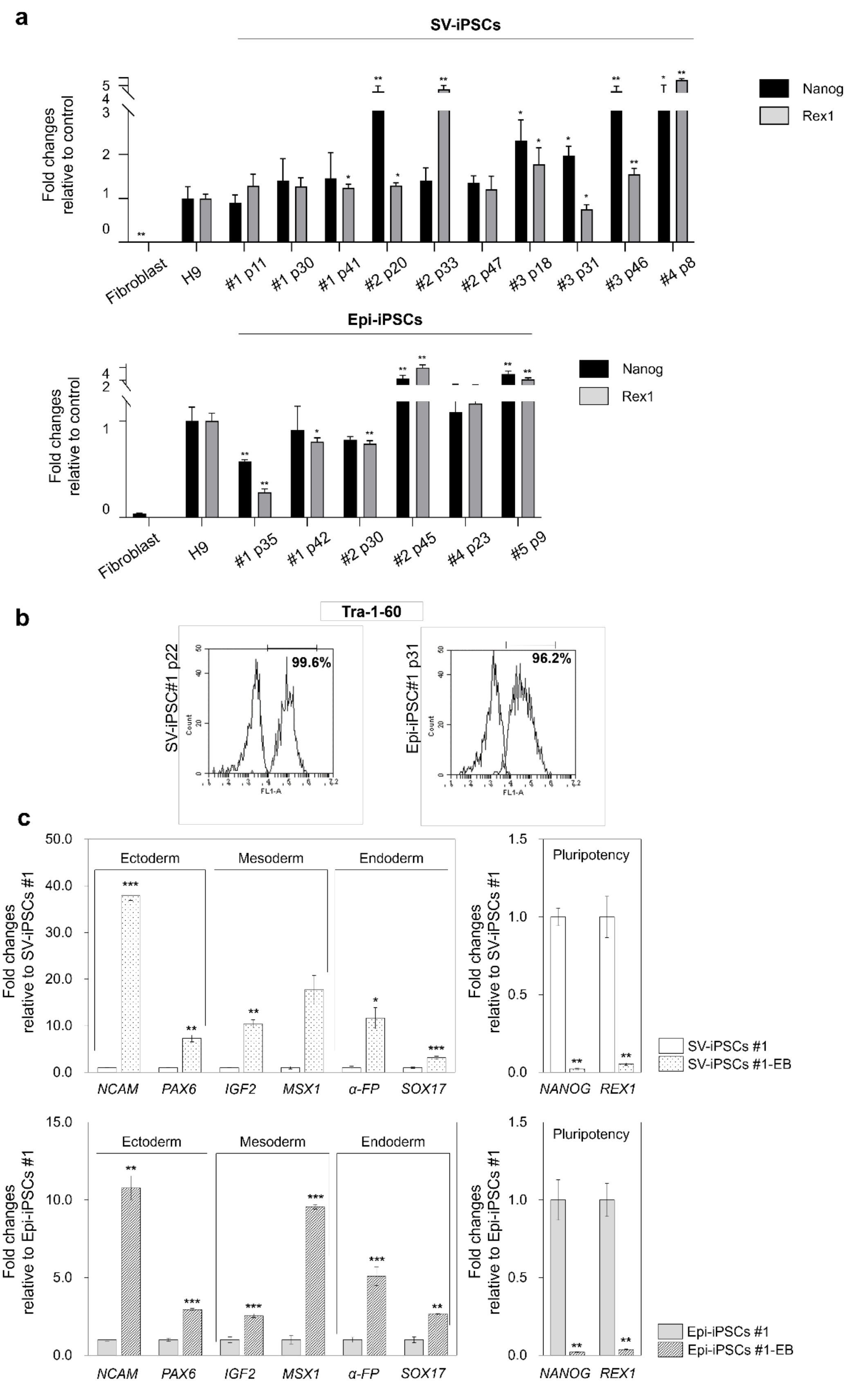
a
SV-iPSCs
Fold changes relative to control
Nanog
Rex1
Fibroblast
H9
#1 p11
#1 p30
#1 p41
#2 p20
#2 p33
#2 p47
#3 p18
#3 p31
#3 p46
#4 p8
Epi-iPSCs
#1 p35
#1 p42
#2 p30
#2 p45
#4 p23
#5 p9
b
Tra-1-60
SV-iPSC#1 p22
99.6%
Epi-iPSC#1 p31
96.2%
Count
FL1-A
c
Ectoderm
Mesoderm
Endoderm
Fold changes relative to SV-iPSCs #1
NCAM
PAX6
IGF2
MSX1
α-FP
SOX17
Pluripotency
NANOG
REX1
SV-iPSCs #1
SV-iPSCs #1-EB
Fold changes relative to Epi-iPSCs #1
Epi-iPSCs #1
Epi-iPSCs #1-EB

Supplementary Figure 2. (a) Relative expression levels of *NANOG* and *REX1* in indicated passages of induced pluripotent stem cells (iPSCs) generated using Sendai virus (SV-iPSC) and episomal vectors (Epi-iPSC), measured by qRT-PCR. Data represent means ± SDs of three independent experiments. $^*P < 0.05$, $^{**}P < 0.01$, compared to control H9 (t-test). (b) Flow cytometric analysis of Tra-1-60 expression in passaged SV-iPSC (p22) and Epi-iPSC (p31). (c) Relative expression levels of markers for the three-germ layers [*NCAM* and *PAX6* (ectoderm), *IGF2* and *MSX1* (mesoderm), *α-FP* and *SOX17* (endoderm)] in *in vitro* differentiated embryoid bodies (EBs) derived from SV-iPSC and Epi-iPSC, measured by qRT-PCR. Data represent means ± SDs of three independent experiments. $^*P < 0.05$, $^{**}P < 0.01$, $^{***}P < 0.001$, compared to control SV-iPSC#1 and Epi-iPSC#1 (t-test).

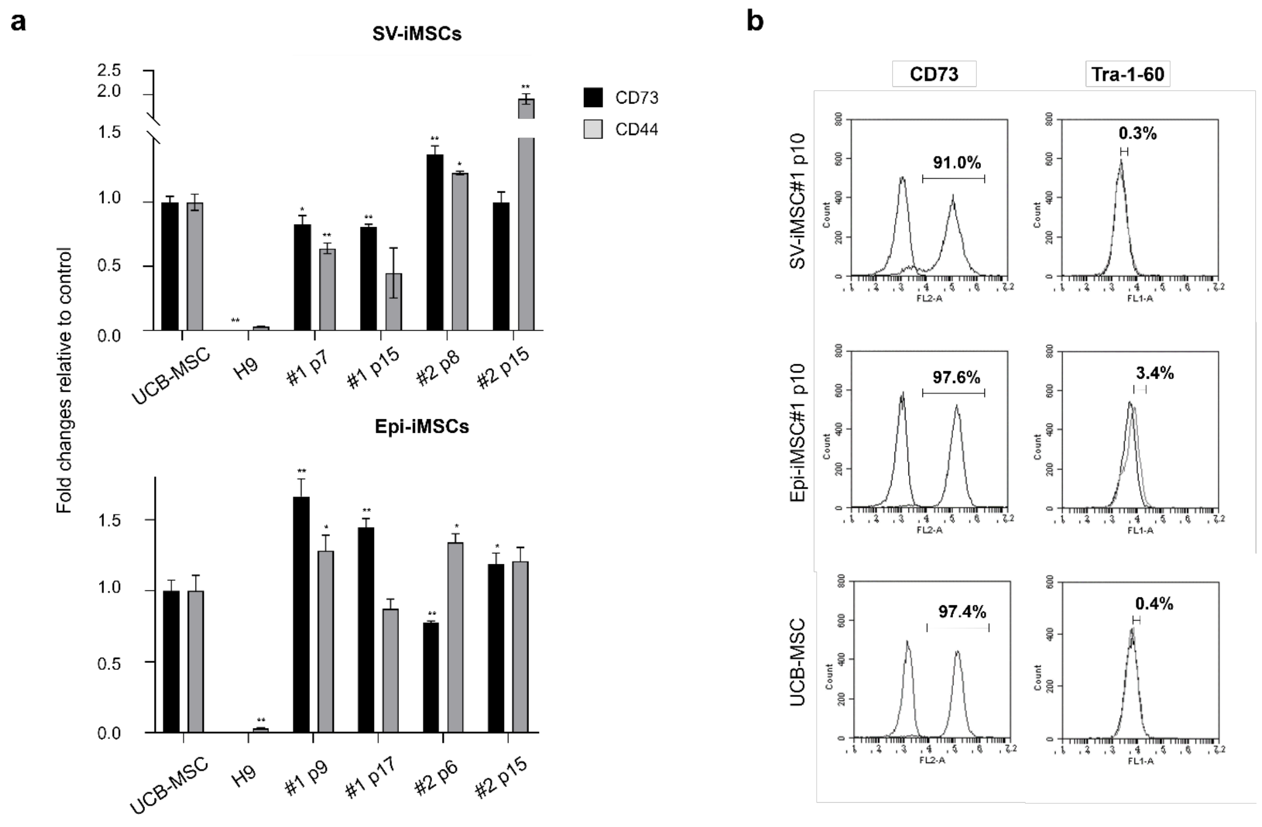

Supplementary Figure 3. (a) Relative expression levels of CD73 and CD44 in indicated passages of induced pluripotent stem cell-derived mesenchymal stem/stromal cells (iMSCs) generated using Sendai virus (SV-iMSC) and episomal vectors (Epi-iMSC), measured by qRT-PCR. Data represent means ± SDs of three independent experiments. *$P < 0.05$, **$P < 0.01$, compared to control umbilical cord blood-derived MSC (UCB-MSC) (t-test). (b) Flow cytometric analysis of CD73 and Tra-1-60 expression in passaged SV-iMSCs (p10) and Epi-iMSCs (p10).

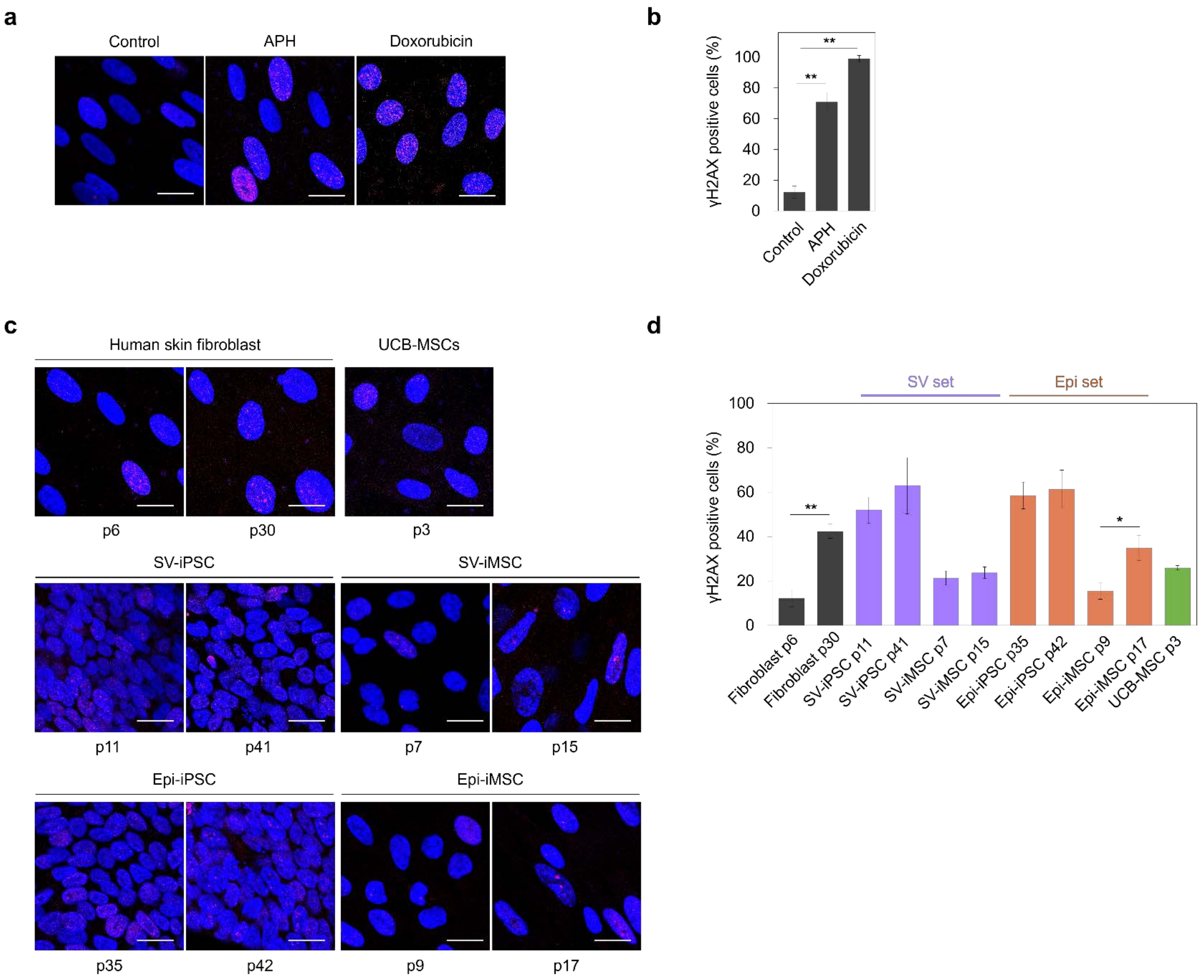
a
Control
APH
Doxorubicin
b
γH2AX positive cells (%)
100
80
60
40
20
0
**
**
Control
APH
Doxorubicin
c
Human skin fibroblast
UCB-MSCs
p6
p30
p3
SV-iPSC
SV-iMSC
p11
p41
p7
p15
Epi-iPSC
Epi-iMSC
p35
p42
p9
p17
d
SV set
Epi set
100
80
60
40
20
0
γH2AX positive cells (%)
**
*
Fibroblast p6
Fibroblast p30
SV-iPSC p11
SV-iPSC p41
SV-iMSC p7
SV-iMSC p15
Epi-iPSC p35
Epi-iPSC p42
Epi-iMSC p9
Epi-iMSC p17
UCB-MSC p3

Supplementary Figure 4. Detection of γH2AX signal by confocal microscopy. (a) Representative images of γH2AX signal in human skin fibroblasts (p6) either untreated (Control) or treated with 0.25 μM aphidicolin (APH) or 2.67 μM doxorubicin for 24 h. (b) Frequency of γH2AX-positive cells in fibroblasts (p6) either untreated (Control) or treated with 0.25 μM APH or 2.67 μM doxorubicin for 24 h. (c) Representative images of γH2AX signal in fibroblasts (p6 and p30), umbilical cord blood-derived mesenchymal stem/stromal cells (UCB-MSC, p3), induced pluripotent stem cells generated using Sendai virus (SV-iPSC, p11 and p41), SV-iPSC-derived MSCs (SV-iMSC, p7 and p15), iPSC generated using episomal vectors (Epi-iPSC, p35 and p42), and Epi-iPSC-derived MSCs (Epi-iMSC, p9 and p17). (d) Frequency of γH2AX-positive cells in fibroblasts (p6 and p30), UCB-MSCs (p3), SV-iPSC (p11 and p41), SV-iMSC (p7 and p15), Epi-iPSC (p35 and p42), and Epi-iMSC (p9 and p17). γH2AX (red), 4′,6-diamidino-2-phenylindole (DAPI, blue). Scale bar = 10 μm. Data represent means ± SDs of three independent experiments. *$P < 0.05$, **$P < 0.01$.

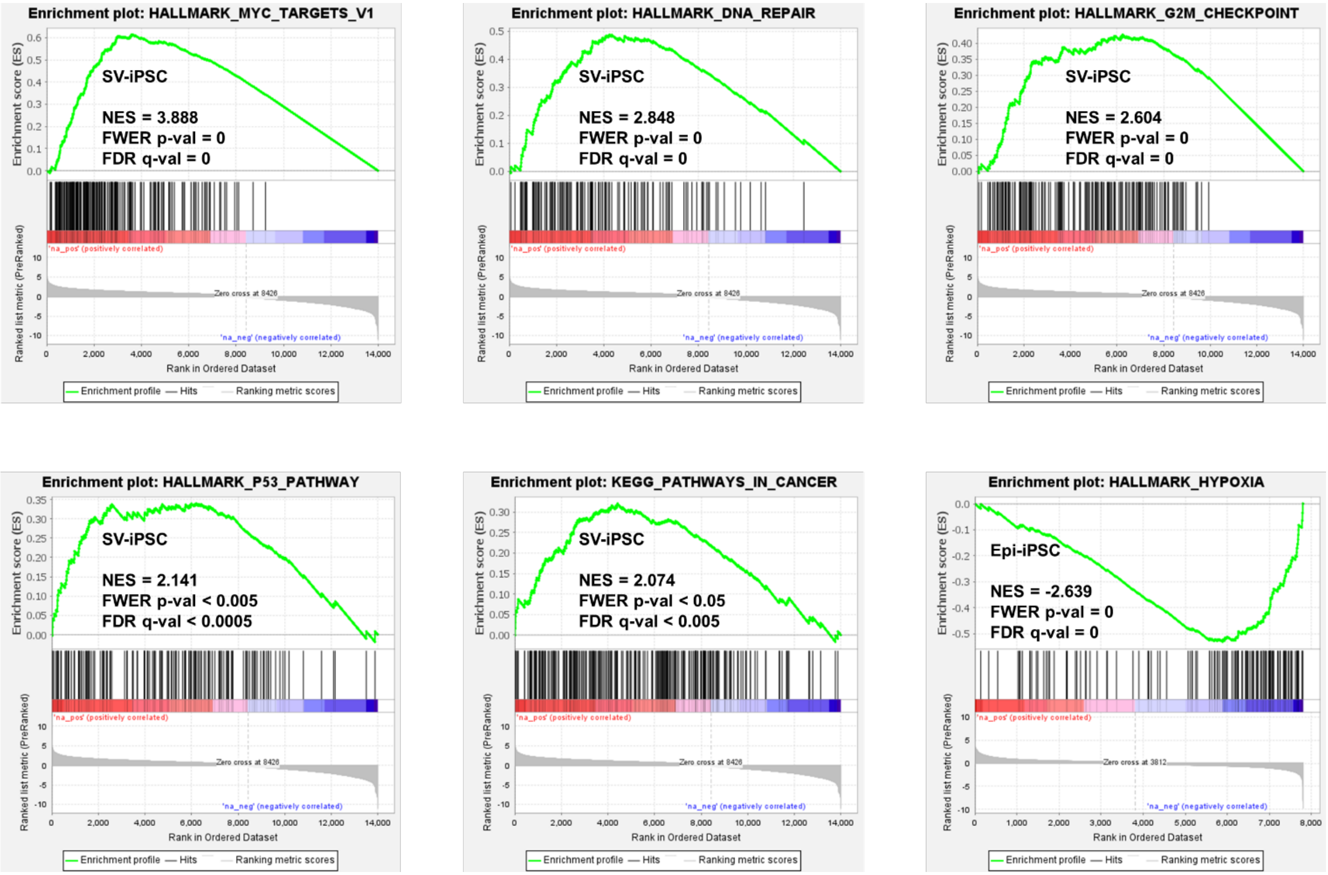

Supplementary Figure 5. Representative enriched pathways in induced pluripotent stem cells (iPSCs) generated using Sendai virus (SV-iPSC) and episomal vectors (Epi-PSC), comparing mid- and late-passage cells: The pathways include MYC targets, DNA repair, G2M checkpoint, p53, Cancer-related pathways, and hypoxia.

Supplementary Table 1. List of antibodies

**Antibody**	**Source**	**Identifier**
Oct3/4 (H-134)	Santa Cruz Biotechnology	Cat. No. sc-9081; RRID: AB_2167703
Nanog	R & D Systems	Cat. No. AF1997; RRID: AB_355097
TRA-1-81	Millipore	Cat. No. MAB4381; RRID: AB_177638
SSEA-3	R & D Systems	Cat. No. MAB1434; RRID: AB_357703
TRA-1-60	Millipore	Cat. No. MAB4360, RRID: AB_2119183
SSEA-4	R & D Systems	Cat. No. MAB1435; RRID: AB_357704
Tuj1	BioLegend	Cat. No. 802001; RRID: AB_2564645
Nestin	Millipore	Cat. No. MAB5326; RRID: AB_2251134
Desmin	Millipore	Cat. No. AB907; RRID: AB_2092609
Actin, alpha-Smooth Muscle (α-SMA)	Sigma-Aldrich	Cat. No. A5228; RRID: AB_262054
HNF3beta/FOXA2	Millipore	Cat. No. 07-633; RRID: AB_390153
Sox17	R & D Systems	Cat. No. MAB1924; RRID: AB_2195646
phospho-Histone H2A.X (Ser139), clone JBW301	Millipore	Cat. No. 05-636; RRID: AB_309864
PE-conjugated CD73	Miltenyi Biotec	Cat. No. 130-095-182; RRID: AB_10829775
PE-conjugated CD105	Miltenyi Biotec	Cat. No. 130-094-941; RRID: AB_10828803
PE-conjugated CD90	Miltenyi Biotec	Cat. No. 130-095-400; RRID: AB_10839701
FITC-conjugated CD34	BioLegend	Cat. No. 343604; RRID: AB_1732005
FITC-conjugated CD45	BioLegend	Cat. No. 304006; RRID: AB_314394
FITC-conjugated HLA-ABC	BioLegend	Cat. No. 311404; RRID: AB_314872
FITC-conjugated HLA-DR	BioLegend	Cat. No. 307604; RRID: AB_314682

Supplementary Table 2. Primers for qRT-PCR

Gene	Forward (5' → 3')	Reverse (5' → 3')
OCN	CAA AGG TGC AGC CTT TGT GTC	TCA CAG TCC GGA TTG AGC TCA
ALP	TAC AAG GTG GTG GGC GGT GAA CGA	TGG CGC AGG GGC ACA GCA GAC
COL1A1	CCT GGA TGC CAT CAA AGT CT	AAT CCA TCG GTC ATG CTC TC
SOX9	AGC GCC CCC ACT TTT GCT CT	GCT CGC CCT TGG GGA ACG TG
COL2A1	CTG GTG ATG ATG GTG AAG	CCT GGA TAA CCT CTG TGA
ACAN	GGA CTT CCG CTG GTC AGA TG	GTT TGT AGG TGG TGG CTG TG
PLIN1	ACC CCC CTG AAA AGA TTG CTT	GAT GGG AAC GCT GAT GCT GTT
LPL	TGG AGG TAC TTT TCA GCC AGG AT	CGT GGG AGC ACT TCA CTA GCT
PPARγ2	TGA ATG TGA AGC CCA TTG AA	CTG CAG TAG CTG CAC GTG TT
NANOG	AATGGTGTGACGCAGGGATG	TGCACCAGGTCTGAGTGTTC
REX1	AATGCGTCATAAGGGGTGAG	TCAATGCCAGGTATTCCTCC
CD73	CGC AAC AAT GGC ACA ATT AC	CAG GTT TTC GGG AAA GAT CA
CD44	AAG GTG GAG CAA ACA CAA CC	AGC TTT TTC TTC TGC CCA CA
NCAM	AGG AGA CAG AAA CGA AGC CA	GGT GTT GGA AAT GCT CTG GT
PAX6	GCC AGC AAC ACA CCT AGT CA	TGT GAG GGC TGT GTC TGT TC
IGF2	CAG ACC CCC AAA TTA TCG TG	GCC AAG AAG GTG AGA AGC AC
MSX1	CGA GAG GAC CCC GTG GAT GCA GAG	GGC GGC CAT CTT CAG CTT CTC CAG
α-FP	ACT GCA ATT GAG AAA CCC ACT GGA GAT G	CGA TGC TGG AGT GGG CTT TTT GTG T
SOX17	CGC TTT CAT GGT GTG GGC TAA GGA CG	TAG TTG GGG TGG TCC TGC ATG TGC TG
GAPDH	GAA GGT GAA GGT CGG AGT C	GAA GAT GGT GAT GGG ATT TC

Supplementary Table 3. List of Online Mendelian Inheritance in Man (OMIM) genes included in identified copy number alterations

Copy number alteration	OMIM Genes
arr[GRCh37] 3q26.31(175356150_175670285) (14 Genes)	*APMR2, FEB10, APMR2, FEB10, TNIK, PLD1, FNDC3B, GHSR, TNFSF10, NCEH1, ECT2, SPATA16, NLGN1, NAALADL2)*
arr[GRCh37] 7q21.11(77809501_79078925) (15 Genes)	*PHTF2, MHS3, GNAI1, CD36, HGF, SEMA3E, GUSB, CACNA2D1, SEMA3C, SEMA3A, PCLO, MAGI2, GNAT3, SEMA3D, GRM3*
arr[GRCh37] 2q21.3q22.1(136610400_137492757) (14 Genes)	*TMEM163, ACMSD, CCNT2, RAB3GAP1, ZRANB3, R3HDM1, MIR128-1, UBXN4, LCT, MCM6, DARS1, CXCR4, HNMT, NXPH2*
arr[GRCh37] 10q21.1(53887679_54013793) (12 Genes)	*PRKG1, MYP15, CSTF2T, DKK1, MBL2, PCDH15, ZWINT, IPMK, CISD1, UBE2D1, TFAM, BICC1*
arr[GRCh37] 3p22.1(42001363_42378111) (27 Genes)	*CCR8, SLC25A38, RPSA, SNORA62, MOBP, MYRIP, ENTPD3, RPL14, CTNNB1, ULK4, TRAK1, CCK, LYZL4, VIPR1, SEC22C, SS18L2, NKTR, ZBTB47, KLHL40, HHATL, HIGD1A, ACKR2, CYP8B1, GASK1A, POMGNT2, SNRK, ANO10*
arr[GRCh37] 1p36.33(1482736_1664221) (45 Genes)	*SAMD11, NOC2L, KLHL17, PERM1, HES4, ISG15, AGRN, MIR200B, MIR200A, MIR429, TNFRSF18, TNFRSF4, SDF4, B3GALT6, C1QTNF12, UBE2J2, SCNN1D, INTS11, CPTP, TAS1R3, DVL1, MXRA8, AURKAIP1, CCNL2, MRPL20, VWA1, ATAD3C, ATAD3B, ATAD3A, TMEM240, SSU72, MIB2, MMP23B, CDK11B, SLC35E2B, MMP23A, CDK11A, NADK, GNB1, CALML6, CFAP74, GABRD, PRKCZ, FAAP20, SKI*
arr[GRCh37] 3q12.2(100445406_100802027) (7 Genes)	*TBC1D23, NIT2, TOMM70, TMEM45A, ADGRG7, TFG, ABI3BP*
arr[GRCh37] 8q22.2(99033353_100013229) (14 Genes)	*MATN2, RPL30, HRSP12, POP1, STK3, KCNS2, OSR2, VPS13B, COX6C, RGS22, FBXO43, POLR2K, SPAG1, RNF19A*
Whole chromosome 8 (843 Genes)	*SPG8, CCNE2, BTF3P12, FGF17, CYP7B1, ZFPM2, RPS20, MSC, LY6H, FOXH1, TNFRSF10D, TNFRSF10C, TNFRSF10B, TNFRSF10A, RECQL4, MTMR7, MYOM2, RIPK2, FZD6, CCN4, SPAG1, TNKS, IKBKB, JRK, ATP6V1C1, GPAA1, RNF139, NSMAF, DLC1, TRPA1, ZHX1, RRM2B, PABPC1, RECQL2, OSGIN2, PDGFRL, TONSL, DEFA3, PLPBP, TRPS1, BROVCA1, RAD54B, PLAG1, CPNE3, RPL8, RPL7, SFRP1, ARFGEF1, HHLA1, TPD52, PNMA2, RP1, EIF3H, BAG4, MED21, GPR20, DEFB4A, EIF3E, UBXN8, RHYNS, SNAI2, MATN2, IMPA1, DEFB1, NKX3-1, SQLE, NCOA2, GFRA2, ANGPT2, SDCBP, KLF10, SLC7A2, HSD17B4, ANGPT1, OC90, EYA1, HAS2, RBPMS, ADAM2, GGH, PNOC, BOS1, CDH17, SCZD6, UBE2V2, HEY1, KCNS2, DUSP4, SCARA3, ADAM9, ADGRB1, NBN, TNFRSF11B, MCM4, DYT6, ASH2L, ANXA13, HRSP12, POP1, PSCA, DPYSL2, EGR3, GML, WWP1, HR, MMP16, KCNQ3, EIF4EBP1, RIMS2, PI15, SLC39A4, HDLCQ2, BRF2, ZFHX4, ATP6V1B2, CHRNA6, TOX, RB1CC1, ADGRA2, HBFQTL4, NCALD, LOXL2, NSD3, DEFB103A, NSFL1C, FBXO32, BAALC, GDAP1, SPAG11B, LZTS1, PINX1, EXOSC4, RNF29, RAD21, TGS1, PTP4A3, CCAR2, CMT1F, SCRIB, EMC2, TCIM, CMT2E, COLEC10, RDH10, SGK3, ENTPD4, EPPK1, KN, MKS3, BIN3, RHOBTB2, KIF13B, LEPROTL1, ADAM7, LSM1, CHRAC1, RGS20, ST3GAL1, MYMY2, RNF19A, MCPH1, TRAM1, EXTL3, OXR1, LYPLA1, FGF20, DLGAP2, DKK4, TMED10, BNIP3L, MFHAS1, TACC1, JPH1, NDRG1, LY96, PAG1, FABP5, CNGB3, STK3, DOK2, SOX5, AURKB, PPP2R2A, CNOT7, TAF2, DGAT1, COPS5, PUF60, POLR2K, ADAMDEC1, TOP1MT, MTMR9, AGO2, LY6D, ADAM28, TP53INP1, FZD3, XPO7, SLURP1, LYNX1, NUDCD1, PKIA, CMT4D, CPSF1, PDP1, HNF4G, ASAP1, DCSTAMP, MDM2BP, STAU2, KCNK9, SCRT1, TEX15, FGL1, EBAG9, IDO1, NEFM, NDNC1, SMAPME, MYBL1, SLC20A2, MSR1, GNRH1, LFS, LMHD, LADD1, KWE, HH2, INSDM, NEFL, IGF1R, IL7, HYPT4, HH7, HCIN, RCC, FCHL3, FHCL1, SDC2, NRG1, HSF1, SLC25A32, GSR, PLAT, FDFT1, SPH1, ODF1, RPL30, RP1, RAB2, SFTPC, ILD2, PTOS1, FGFR2, PPP2CB, POLB, GRINA, PDE7A, PEX2, PMP2, OTFCS, OGD, GLI4, OPRK1, PVT1, LYN, CCN3, OPDM1, CLN4, PPP3CC, COL14A1, CHRNB3, CHRNA2, CYP7A1, KFS1, CEBPD, CTSB, CA8, CA1, CA3, CRC, CR1, CALB1, BL, BRCA1, BOR1, BMP1, ADRB3, NAT1, ADRA1A, HALD1, ADCY8, SCS, EEF1D, GPT, GLM1, GABRR2, GFUS, FGFR1, FNTA, EXT1, RUNX1T1, EPHX2, EBS5A, PENK, AI3A, CLU, T2D, EPB49, DEFA1, DDOD, COX6C, CYP11B2, CYC1, BSTVS, JWS, CRH, BFNS2, HNSCC, DEFA5, DEFA6, FABP4, TTPA, PCM1, GEM, CLN8, CCT3,*

	SNTB1, GRPR, WRN, AVED, GATA4, TDH3, SEMDSP, SPG5A, IGF1RES, RTS2, RCDFRD, RBS, RP, ACHM3, CPP, SLA, NDUFB9, TCEA1, KAT6A, TUSC3, LY6E, YWHAZ, PLEC, ZNF16, MSRA, PTK2B, STC1, DEFA4, GDF6, OPLAHD, FAME1, ENPP2, RHPN1, E2F5, TERF1, PRKDC, ELOC, PTK2, NPBWR1, STMN2, STAR, ASPH, BLK, CHEGDD, APL, ABS2, ALUNC, ADCC, LCAH, AEZ, ZNF7, ZNF34, SLC18A1, UQCRB, IBGC1, TP53, TRIGNO1, TRPS3, TRPS1, MYC, MOS, GTF2E2, TRHR, TG, TOF, POLR3D, SYT1, JEB5B, OPTB3, MPS3C, NBS, MCPH1, 3MC3, MDM, PDB5, PRLTS1, FRBRL, KCNB2, EBS5B, DPYSD, DECR1, RTSC1, BGS, CTHM, COH1, COACH1, JHS, CMT4A, ARHGAP39, ARTHS, DCAF13, FAM86B2, FAM86B1, MDDGC12, SRXY9, PCAT1, ECTDS, BBS14, IMD26, PXDNL, NDUFAF7, FSBP, DIAR7, NUDT18, POU5F1B, RHDA2, KIAA1456, RGS22, JBTS21, NEDFCF, IMD15B, VRJS, NRBP2, TACHD, MRT39, DOCK5, KCTD9, SLC7A13, SCKL10, NSMCE2, ST18, ZNF703, GSDMD, HMNDYT2, TRIM35, C8ORF17, TTD6, FUT10, MTERF3, CILD28, RREI, LAMSHF, SNORD13, CYHR1, CSGALNACT1, TMEM65, RP73, EBS5D, UNC5D, ZLS2, VIRMA, PYCRL, CFAP418, HH12, AGPAT5, TMEM66, BCC7, TSPYL5, BVVLS2, COXPD10, CDLS4, MTO1, RNF170, RALYL, BBDS1, CORD16, OI13, PSD3, AVSD4, VSD1, TTI2, 11-Feb, ETL5, HAS2AS1, PNCA4, UBE2W, OPLAH, MIR320A, MC3DN3, HRTFDS, MC3DN6, PRNCR1, SPIDR, LCA17, HH20, MDDGA12, POMK, KIFC2, KCNU1, PLEKHF2, SLC35G5, TDH, CIBAR1, PRSS55, LY6K, FEPS1, SPG54, DDHD2, SH2D4A, CILD19, LRRC6, SNX16, SPG53, PBD5B, PBD5A, CRSDS, CCAT2, LMPHM10, LRRC14, TATDN1, HAE5, HRURF, LESKRES, CCDC25, NUGGC, TDRP, MIR30D, MIR30B, ENY2, MTFR1, COXPD47, IQANK1, FABP12, LACTB2, FRTS5, SRS4, SDJLABA, MYP27, RP88, KBTBD11, CDCA2, CPQ, YTHDF3, IDDMDS, DCAF4L2, PSKH2, ARMC1, OZEMA20, LETM2, SAIDV, MFSD3, TMEM71, FHIP2B, SPGF79, MIR151A, PCMTD1, PHF20L1, DEDISB, SNX31, EPDR1, OZEMA12, SPGF64, SPG85, ALKAL1, ODG9, SLC45A4, CLXN, ZNF706, ADAM18, CASC19, TETAMS2, DFNB109, DEE64, CYRIB, SPGF25, SYNS4, FANCS, CMTDIG, GPIBD15, LRRCC1, PPP1R42, CCAT1, CASC11, HHRRD, CASC21, CASC8, PCAT2, MCMDC2, DENND3, IDDFSDA, PTCSC1, ISDNA, BBS21, ANXD2, PRAG1, MRT59, EPEO1, CMT1G, ZC3H3, ZDHHC2, MAPK15, HMBOX1, ADAM32, SLC10A5, ZBTB10, CHNG7, NEDCHS, DUSP26, RRS1, EV5, LRP12, MC5DN2, SEC11A, MC1DN24, MC1DN17, PAPA9, BAGOS, IMD15A, BMFS5, SQSD, SAMD12, ATP6V0D2, PURG, PPCD4, PDLIM2, USP17L2, FAM167A, TM2D2, VDAC3, COL22A1, SULF1, IMD54, EBF2, VPS37A, TMEM67, PIP4P2, ABRA, DIH3, LPL, MAL2, MTUS1, PARP10, CPA6, LINC00293, THAP1, ZNF395, FAM84B, TRIB1, GOLGA7, ESCO2, RSPO2, SORBS3, NKX6-3, HTRA4, JBTS6, UBXN2B, WASHC5, CTHRC1, MICU3, PLPP5, CYP11B1, GINS4, BOP1, REEP4, PPP1R3B, AP3M2, HGSNAT, KHDRBS3, SLC25A37, ENFL4, MTDH, PIWIL2, MAFA, MED30, MAF1, MRPL13, AGPAT6, MTSS1, SLC26A7, UBR5, CSMD3, CSMD1, GSDMC, CMTRIA, LGI3, SNCV, COMMD5, EXT1, AITD3, KCNV1, PHYHIP, ARHGEF10, SGCZ, NPM2, NETO2, ASD2, AZIN1, SLC52A2, PKHD1L1, CLN8, CMT2K, HOOK3, VPS13B, PLEKHA2, CHD7, MIR124-1, CLDN23, ZHX2, PPP1R16A, FBXO43, FBXO25, FBXL6, SCX, SNAX1, NEIL2, POTEA, SPATC1, RBM45, ATP6V1H, DERL1, PDHPD, ERI1, RAB11FIP1, SLC39A14, SNTG1, DFNA28, RP1L1, GRHL2, FBXO16, ECCL, FAM90A18, FAM90A15, FAM90A14, FAM90A13, FAM90A12, FAM90A10, FAM90A9, FAM90A8, FAM90A7, FAM90A5, FAM90A3, CCDC26, GLM7, FAM90A19, DEPDC6, DCTN6, ESRP1, NKAIN3, PTDSS1, CORD9, GPIHBP1, ELP3, ANK1, PEBP4, ARC, TMEM70, NDUFAF6, SLCO5A1, IMPAD1, DNAJC5B, TMEM74, DPY19L4, CBAS3, RNF213, LGMDR17, MIR661, MCOPCB6, VUR3, OCMD, NPHP11, HH5, BHLHE22, ASAH1, MODY11, DPYS, LAPTM4B, SCAR34, DSCC1, MRT13, MCOP4, PEOA5, ALL, FAM90A20, CHCHD7, SYBU, NAPRT, CA2, CA13, FAM110B, MGS, INTS10, INTS9, INTS8, SCARA5, OSR2, CLVS1, TRMT12, ERLIN2, SPG18B, PBK, DNAJC5, SLC30A8, CHMP7, MRT7, ADHFE1, ZFAT1, SOX17, SMDP2, CHMP4C, ATAD2, BIBARS, SNORD87, SNHG6, SOX7, NAT2, PREX2, EBS5C, IDO2, MTDPS8A, OTUD6B, MRPS28, TRAPPC9, VPS28, TMEM64, MBOAT4, FAM83H, SHARPIN, RMDN1, MRPL15, MRPL9, EFR3A, PRDM14, NKX2-6, VCPIP1, SEPTIN10, CSPP1
arr[GRCh37] 3p14.21(59596931_65725694) (7 Genes)	*FAM107A, FAM3D, FHIT, PTPRG, FEZF2, CADPS, SNTN*

Supplementary Table 4. Gene expression changes in 25 chromosome instability genes: log2 ratios of each gene and *P*-value comparisons between mid- and late-passage induced pluripotent stem cell generated using Sendai virus (SV-iPSC) and episomal vectors (Epi-iPSC).

List	Gene	Ensemble ID	SV-iPSC log2	SV-iPSC *P*-value	Epi-iPSC log2	Epi-iPSC *P*-value
AURKA (Aurora Kinase A)	*AURKA*	ENSG00000087586	1.911637151	0.0000	-0.161546954	0.1837
BIRC5 (Baculoviral IAP Repeat Containing 5, also known as Survivin)	*BIRC5*	ENSG00000089685	2.091883	0.0000	-0.640341002	0.0000
BUB1 (Budding Uninhibited by Benzimidazoles 1 Homolog)	*BUB1*	ENSG00000169679	1.31739665	0.0000	-0.149722896	0.1991
CDC2 (Cell Division Cycle 2, also known as *CDK1*)	*CDK1*	ENSG00000170312	1.377426705	0.0000	-0.692779509	0.0000
CDC20 (Cell Division Cycle 20)	*CDC20*	ENSG00000117399	2.133711887	0.0000	0.081130737	0.5011
CDC25C (Cell Division Cycle 25C)	*CDC25C*	ENSG00000158402	1.086347008	0.0000	-0.216566266	0.1087
CDCA8 (Cell Division Cycle Associated 8, also known as Borealin)	*CDCA8*	ENSG00000134690	1.801523792	0.0000	-0.146123785	0.2510
CENPF (Centromere Protein F)	*CENPF*	ENSG00000117724	-0.264967974	0.0222	0.121671661	0.2899
CENPA (Centromere Protein A)	*CENPA*	ENSG00000115163	1.530216702	0.0000	-0.239240235	0.0869
CEP55 (Centrosomal Protein 55)	*CEP55*	ENSG00000138180	0.854767868	0.0000	0.342530876	0.0119
DLGAP5 (Discs, Large Homolog-Associated Protein 5, also known as *HURP*)	*DLGAP5*	ENSG00000126787	1.406288144	0.0000	-0.167019312	0.1556
KIF2C (Kinesin Family Member 2C, also known as *MCAK*)	*KIF2C*	ENSG00000142945	1.602446667	0.0000	-0.439978639	0.0003
KIF4A (Kinesin Family Member 4A)	*KIF4A*	ENSG00000090889	0.926207558	0.0000	-0.258008953	0.0351
KIF11 (Kinesin Family Member 11, also known as Eg5)	*KIF11*	ENSG00000138160	1.398370856	0.0000	-0.018754171	0.8734
MELK (Maternal Embryonic Leucine Zipper Kinase)	*MELK*	ENSG00000165304	1.188776218	0.0000	0.081109403	0.5125
NDC80 (NDC80 Kinetochore Complex Component)	*NDC80*	ENSG00000080986	0.946050614	0.0000	-0.104885502	0.4042
NEK2 (NIMA-Related Kinase 2)	*NEK2*	ENSG00000117650	1.601147475	0.0000	-0.494249091	0.0003
PLK1 (Polo-Like Kinase 1)	*PLK1*	ENSG00000166851	1.943399631	0.0000	0.173886762	0.1437
PRC1 (Protein Regulator of Cytokinesis 1)	*PRC1*	ENSG00000198901	0.989144057	0.0000	-0.116587455	0.3489
SPC25 (SPC25 Component of NDC80 Kinetochore Complex)	*SPC25*	ENSG00000152253	1.157019093	0.0000	0.311118126	0.0299
TOP2A (Topoisomerase (DNA) II Alpha)	*TOP2A*	ENSG00000131747	1.141895933	0.0000	-0.588238144	0.0000
TTK (TTK Protein Kinase, also known as *MPS1*)	*TTK*	ENSG00000112742	1.465914334	0.0000	-0.188700312	0.1166
UBE2C (Ubiquitin-Conjugating Enzyme *E2C*)	*UBE2C*	ENSG00000175063	2.381088673	0.0000	-0.601858206	0.0000
ZWILCH (ZW10 Interacting Kinetochore Protein Homolog)	*ZWILCH*	ENSG00000174442	1.868682191	0.0000	0.187710251	0.1250
ZWINT (ZW10 Interacting Kinetochore Protein)	*ZWINT*	ENSG00000122952	0.636528691	0.0000	0.221979125	0.0790

Supplementary Table 5. The Gene Set Enrichment Analysis (GSEA) of overrepresented hallmark and Kyoto encyclopedia of genes and genomes (KEGG) pathways comparing mid- and late-passage induced pluripotent stem cell generated using Sendai virus (SV-iPSC) and episomal vectors (Epi-iPSC).

Cell line	Pathway	SIZE	NES	NOM p-val	FDR q-val	FWER p-val
SV-iPSC	HALLMARK_MYC_TARGETS_V1	193	3.8880162	0	0	0
SV-iPSC	HALLMARK_OXIDATIVE_PHOSPHORYLATION	190	3.8828516	0	0	0
SV-iPSC	HALLMARK_E2F_TARGETS	173	3.0847282	0	0	0
SV-iPSC	HALLMARK_DNA_REPAIR	129	2.8476584	0	0	0
SV-iPSC	HALLMARK_MTORC1_SIGNALING	167	2.7980564	0	0	0
SV-iPSC	HALLMARK_REACTIVE_OXYGEN_SPECIES_PATHWAY	42	2.7781022	0	0	0
SV-iPSC	HALLMARK_TNFA_SIGNALING_VIA_NFKB	137	2.7526414	0	0	0
SV-iPSC	HALLMARK_FATTY_ACID_METABOLISM	116	2.6991253	0	0	0
SV-iPSC	HALLMARK_PROTEIN_SECRETION	79	2.679755	0	0	0
SV-iPSC	HALLMARK_ADIPOGENESIS	163	2.6617274	0	0	0
SV-iPSC	HALLMARK_G2M_CHECKPOINT	174	2.6039937	0	0	0
SV-iPSC	HALLMARK_UNFOLDED_PROTEIN_RESPONSE	99	2.557647	0	0	0
SV-iPSC	HALLMARK_TGF_BETA_SIGNALING	48	2.4700587	0	0	0
SV-iPSC	HALLMARK_ESTROGEN_RESPONSE_LATE	147	2.3886638	0	0	0
SV-iPSC	HALLMARK_APOPTOSIS	120	2.355609	0	0	0
SV-iPSC	HALLMARK_INTERFERON_ALPHA_RESPONSE	70	2.3220987	0	0	0
SV-iPSC	HALLMARK_ALLOGRAFT_REJECTION	92	2.193932	0	0.000239	0.001
SV-iPSC	HALLMARK_ANDROGEN_RESPONSE	75	2.2107284	0	0.000253	0.001
SV-iPSC	HALLMARK_PI3K_AKT_MTOR_SIGNALING	79	2.2147372	0	0.000267	0.001
SV-iPSC	HALLMARK_P53_PATHWAY	155	2.1407704	0	0.000455	0.002
SV-iPSC	HALLMARK_HYPOXIA	144	2.0412803	0	0.000505	0.003
SV-iPSC	HALLMARK_XENOBIOTIC_METABOLISM	131	2.0575829	0	0.000523	0.003

SV-iPSC	HALLMARK_APICAL_JUNCTION	133	2.074803	0	0.000542	0.003
SV-iPSC	HALLMARK_PEROXISOME	75	2.0805268	0	0.000563	0.003
SV-iPSC	HALLMARK_HEME_METABOLISM	141	2.083084	0	0.000586	0.003
SV-iPSC	HALLMARK_MYC_TARGETS_V2	52	2.0888262	0	0.00061	0.003
SV-iPSC	HALLMARK_INTERFERON_GAMMA_RESPONSE	131	2.0969179	0	0.000637	0.003
SV-iPSC	HALLMARK_UV_RESPONSE_UP	128	2.102826	0	0.000666	0.003
SV-iPSC	HALLMARK_ESTROGEN_RESPONSE_EARLY	149	2.1123796	0	0.000697	0.003
SV-iPSC	HALLMARK_SPERMATOGENESIS	87	1.9993664	0	0.000697	0.004
SV-iPSC	HALLMARK_EPITHELIAL_MESENCHYMAL_TRANSITION	146	1.9892157	0	0.001212	0.005
SV-iPSC	HALLMARK_IL2_STAT5_SIGNALING	129	1.8832417	0	0.002017	0.01
SV-iPSC	HALLMARK_GLYCOLYSIS	159	1.8847111	0	0.00208	0.01
SV-iPSC	HALLMARK_INFLAMMATORY_RESPONSE	104	1.8597877	0	0.002254	0.012
SV-iPSC	HALLMARK_KRAS_SIGNALING_UP	115	1.8435845	0	0.002498	0.014
SV-iPSC	HALLMARK_WNT_BETA_CATENIN_SIGNALING	34	1.8203673	0.010380623	0.003009	0.018
SV-iPSC	HALLMARK_COMPLEMENT	131	1.8102245	0	0.003078	0.019
SV-iPSC	KEGG_RIBOSOME	85	4.157893	0	0	0
SV-iPSC	KEGG_SYSTEMIC_LUPUS_ERYTHEMATOSUS	86	4.1363163	0	0	0
SV-iPSC	KEGG_HUNTINGTONS_DISEASE	147	3.1183062	0	0	0
SV-iPSC	KEGG_PROTEASOME	39	3.0303526	0	0	0
SV-iPSC	KEGG_PARKINSONS_DISEASE	110	2.9925058	0	0	0
SV-iPSC	KEGG_OXIDATIVE_PHOSPHORYLATION	109	2.8639076	0	0	0
SV-iPSC	KEGG_ALZHEIMERS_DISEASE	136	2.8366044	0	0	0
SV-iPSC	KEGG_SPLICEOSOME	109	2.7775915	0	0	0
SV-iPSC	KEGG_CELL_CYCLE	106	2.4054554	0	0.000393	0.001
SV-iPSC	KEGG_CITRATE_CYCLE_TCA_CYCLE	26	2.244131	0	0.000496	0.002
SV-iPSC	KEGG_GLUTATHIONE_METABOLISM	34	2.2512612	0	0.000538	0.002

SV-iPSC	KEGG_BUTANOATE_METABOLISM	22	2.340297	0	0.000587	0.002
SV-iPSC	KEGG_UBIQUITIN_MEDIATED_PROTEOLYSIS	106	2.3616571	0	0.000645	0.002
SV-iPSC	KEGG_TGF_BETA_SIGNALING_PATHWAY	64	2.1854084	0	0.001546	0.007
SV-iPSC	KEGG_VALINE_LEUCINE_AND_ISOLEUCINE_DEGRADATION	34	2.1657977	0	0.001621	0.008
SV-iPSC	KEGG_ALANINE_ASPARTATE_AND_GLUTAMATE_METABOLISM	18	2.127946	0.002932551	0.002356	0.012
SV-iPSC	KEGG_PATHOGENIC_ESCHERICHIA_COLI_INFECTION	45	2.1205013	0	0.002562	0.014
SV-iPSC	KEGG_ENDOCYTOSIS	127	2.0725503	0	0.00381	0.025
SV-iPSC	KEGG_PATHWAYS_IN_CANCER	235	2.073874	0	0.004011	0.025
SV-iPSC	KEGG_P53_SIGNALING_PATHWAY	56	2.0778356	0.004651163	0.004234	0.025
SV-iPSC	KEGG_OOCYTE_MEIOSIS	86	2.0093977	0	0.005475	0.037
SV-iPSC	KEGG_AMINOACYL_TRNA_BIOSYNTHESIS	39	1.9935032	0	0.006291	0.044
SV-iPSC	KEGG_WNT_SIGNALING_PATHWAY	109	1.9906304	0	0.006156	0.045
SV-iPSC	KEGG_OLFACTORY_TRANSDUCTION	62	-3.3024347	0	0	0
SV-iPSC	KEGG_TASTE_TRANSDUCTION	35	-2.273478	0	0	0
Epi-iPSC	HALLMARK_INFLAMMATORY_RESPONSE	85	1.8350023	0	0.046387	0.048
Epi-iPSC	HALLMARK_HYPOXIA	130	-2.6389837	0	0	0
Epi-iPSC	HALLMARK_GLYCOLYSIS	116	-1.859273	0	0.022832	0.042

**Abbreviations**	**Definition**
SIZE	Set Size
NES	Normalized Enrichment Score
NOM p-val	Nominal p-value
FDR q-val	False Discovery Rate q-value
FWER p-val	Family-Wise Error Rate p-value
